# Beyond Junk DNA: Emerging Roles of Non-Coding RNAs in Cancer and Therapeutic Innovation

**DOI:** 10.3390/biom16081110

**Published:** 2026-07-29

**Authors:** Li Qiu, Zenan Xu, Jianxiong Xu, Zhiming Lv, Wenfang Li

**Affiliations:** 1 School of Pharmaceutical Sciences & Institute of Materia Medica, Xinjiang University, Urumqi 830017, China; qiuli@bbmu.edu.cn (L.Q.); xuzenan@stu.xju.edu.cn (Z.X.); jianxiongxu@stu.xju.edu.cn (J.X.); 2 Department of Pharmacy, Anhui Engineering Technology Research Center of Biochemical Pharmaceutical, Bengbu Medical University, 2600 Donghai Avenue, Bengbu 233030, China; 3 Department of Gastrointestinal Surgery, The Fifth Affiliated Hospital of Xinjiang Medical University, Urumqi 830017, China; 4 College of Life Science and Technology, Xinjiang University, Urumqi 830017, China

**Keywords:** junk DNA, non-coding RNAs, cancer, molecular mechanisms, therapeutic targets

## Abstract

Non-coding RNAs (ncRNAs) are the biggest class of regulatory molecules in the human genome, which constitute ~98% of total transcripts, and were once considered as “transcriptional noise”. This review will focus on the newly identified functions of non-coding RNAs (ncRNAs) in cancer biology and therapeutic innovations. We review the existing knowledge on microRNAs, long non-coding RNAs, and circular RNAs in the context of their different experimental and computational studies, and we highlight their mechanisms of action in cancer hallmarks such as proliferation and resistance to apoptosis, metastasis, and metabolic reprogramming. We evaluate the translational landscape of ncRNA-based therapeutics by examining clinical trials in progress and completed, delivery methods, and emerging technologies. This review aims to separate fact from fiction and offer a balanced view on the facts, which will help guide future research and clinical development in the field of ncRNA oncology.

## 1. Introduction

**Figure 1:**
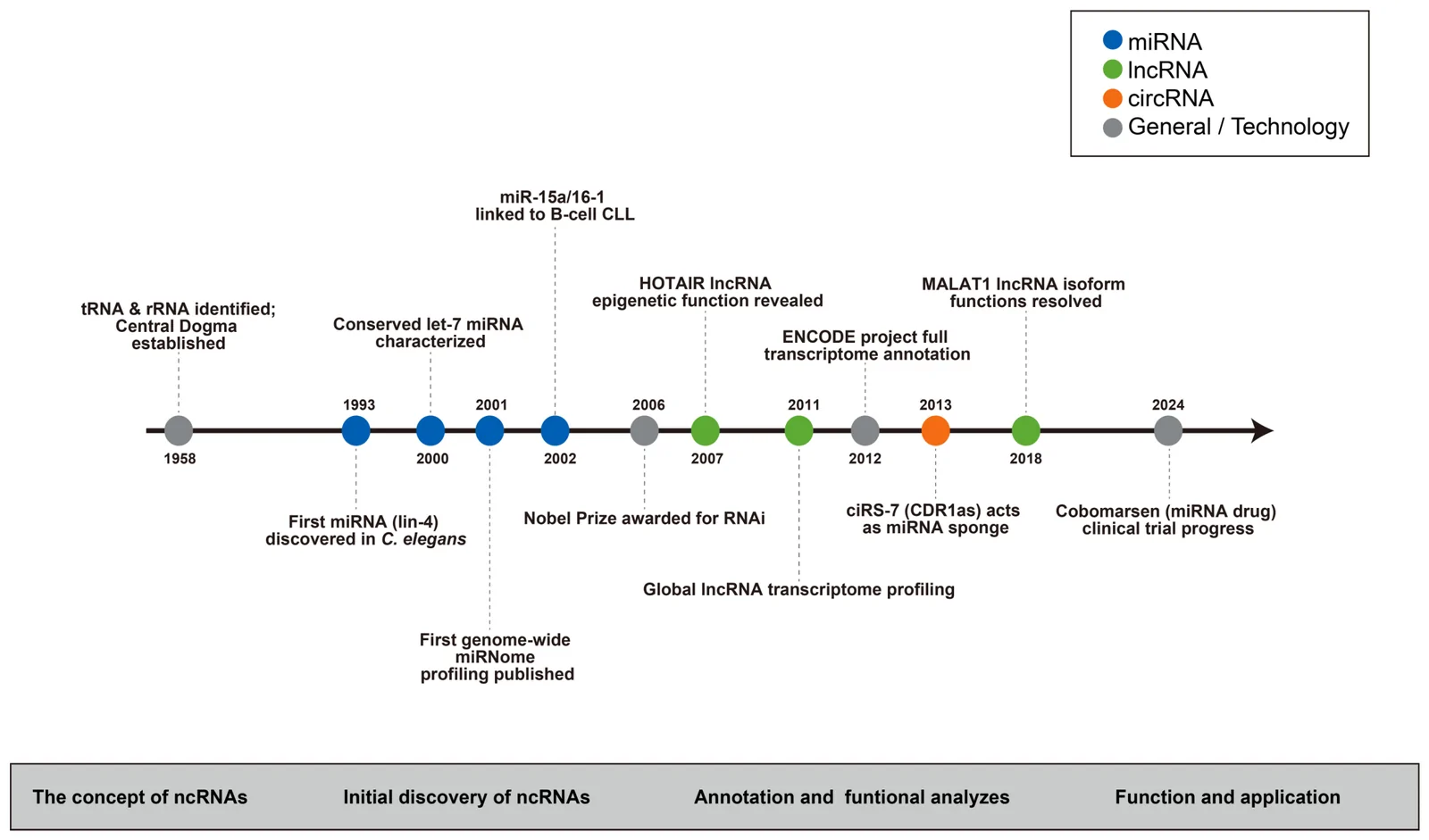
Timeline of landmark discoveries in non-coding RNA research. Key milestones are listed in chronological order and colored by the class of RNA: miRNAs (blue), lncRNAs (green), circRNAs (orange), general/structural RNAs (gray). Only discoveries that have been validated by independent studies are included. Selected milestones: 1958, discovery of functional tRNA/rRNA; 1993, discovery of the first miRNA (lin-4); 2000, discovery of the first miRNA (let-7); 2001, human miRnome; 2002, deletion of miR-15a/16-1 in CLL; 2006, Nobel Prize for RNAi; 2007, discovery of HOTAIR; 2011, pan-cancer profiling of lncRNAs; 2012, ENCODE project; 2013, first functional characterization of circRNA (ciRS-7); 2018, subtype-specific function of MALAT1; 2024, Phase I/II trials of cobomarsen.

Next-generation sequencing (NGS) technology has revealed that, although up to 90% of eukaryotic genomes are transcribed, only 1.5% to 2.0% of transcripts encode proteins, with the majority being non-coding RNAs (ncRNAs) [1]. Early skepticism regarding the functional relevance of ncRNAs was rooted in the “transcriptional noise” hypothesis, which posited that most non-coding transcripts represented functionless byproducts of genomic transcription [2]. However, subsequent evidence has firmly established ncRNAs as essential regulators of diverse cellular processes. Importantly, ncRNAs are classified based on their length, genomic location, and functional mechanisms. Small ncRNAs (<200 nt), including miRNAs, siRNAs, and piRNAs, mediate post-transcriptional gene silencing, whereas long non-coding RNAs (lncRNAs; >200 nt) and circular RNAs (circRNAs) participate in diverse regulatory networks through mechanisms involving chromatin remodeling, transcriptional modulation, and protein sequestration [3]. lncRNAs exert their regulatory functions through multiple molecular mechanisms, including chromatin modification, transcriptional interference, and post-transcriptional processing, operating as signals, decoys, guides, and scaffolds to orchestrate gene expression programs [4]. Structural ncRNAs encompass ribosomal RNA (rRNA), transfer RNA (tRNA), telomeric RNA, small nuclear RNAs (snRNAs), and small nucleolar RNAs (snoRNAs), which are involved in protein synthesis, telomere protection, mRNA precursor processing, and rRNA processing, respectively. Regulatory ncRNAs encompass microRNAs (miRNAs), piwi-interacting RNAs (piRNAs), short interfering RNAs (siRNAs), circular RNAs (circRNAs), and lncRNAs [5], with piRNAs being exclusively found in animals [6]. Regulatory ncRNAs are extensively involved in cellular physiological events, including epigenetic regulation, direct transcriptional control, miRNA sponge activity, mRNA stability modulation, and protein interactions [7]. Abnormal expression of these regulatory ncRNAs has closely been associated with disease onset and progression, often located at “fragile sites” or disease-causing gene loci within the genome [8]. Deletions or rearrangements frequently observed in cancer cells and specific neurological disease suggest their potential as novel drug targets for disease treatment [9]. Monitoring and intervening in abnormal ncRNA genes and their expression levels hold significant promise for diagnosing and treating various genetic, immune, metabolic, and tumor-related diseases [10]. Ferroptosis, an iron-dependent form of regulated cell death driven by lipid peroxidation, represents an emerging target for cancer therapy [11]. ncRNAs have been shown to modulate ferroptosis by regulating key genes involved in iron metabolism, lipid peroxidation, and antioxidant defense, including SLC7A11, GPX4, and ACSL4 [12]. Therefore, it is essential to precisely detect and intervene abnormal miRNAs, circRNAs, and lncRNAs to improve the diagnosis and treatment of ncRNA associated disease. The discovery of the first functional non-coding RNA has led to transformative advances in the field, which have changed our understanding of gene regulation [7]. Every step from the characterization of transfer RNAs and ribosomal RNAs to the discovery of master regulatory roles for miRNAs, lncRNAs, and circRNAs has uncovered another layer of regulatory complexity [13]. Figure 1 shows the timeline of important discoveries in the field of ncRNA research. Together, these advances have revealed that the non-coding genome is not “junk” but a complex regulatory system that plays a critical role in development, homeostasis and disease.

The ncRNAs have unique structural characteristics that enable their various regulatory roles: miRNAs are expressed as conserved hairpin precursors, circRNAs are covalently closed circles that are resistant to exonucleases, and lncRNAs fold into complex secondary structures such as G-quadruplexes and triple helices [14]. While early ncRNA research focused primarily on small RNAs (siRNAs and miRNAs), the past decade has seen an explosive growth in lncRNA and circRNA studies, with over 40,000 human lncRNAs and more than 100,000 circRNAs identified to date [15]. In addition, lncRNAs fold into dynamic and flexible conformations via local and long-range nucleotide pairing, yielding structures such as helices, terminal/internal loops, junctions, pseudoknots, triple-helices, G-quadruplexes, and other advanced structures [16]. Technological advances such as single-cell RNA-seq and spatial transcriptomics have further revealed the cell type-specific and subcellular localization-dependent functions of ncRNAs, challenging previous one-dimensional models of gene regulation [10]. 

The field of lncRNA research has evolved significantly since the discovery of the first eukaryotic lncRNA, H19, in 1984, initially mistaken for an mRNA, in fetal tissues such as the liver, gut, heart, and skeletal *muscle of mice* (*Mus musculus*) [17]. In 1991, Xist, the first functional lncRNA to be discovered, mediates X-chromosome inactivation through the recruitment of chromatin-modifying complexes, establishing a paradigm for lncRNA-mediated epigenetic regulation [18,19]. Concurrently, the steroid receptor RNA activator (SRA) was identified as a functional lncRNA that coactivates nuclear receptors, with subsequent studies highlighting its association with breast cancer progression and therapeutic potential [20]. In 2003, MALAT-1 was identified as a metastatic predictor in non-small cell lung cancer, with its high expression significantly correlating with poor patient prognosis [21]. In 2007, ANRIL (antisense non-coding RNA in the INK4 locus) recruits polycomb repressive complexes PRC1 and PRC2 to the INK4b/ARF/INK4a locus, mediating epigenetic silencing of tumor suppressor genes and promoting cancer cell proliferation [22,23]. In 2008, HOTAIR was found to drive breast cancer metastasis by binding to both polycomb repressive complex 2 (PRC2) and LSD1 complexes to remodel chromatin states [24]. In 2009, multiple studies demonstrated the involvement of PRC2-interacting lncRNAs, including TUG1, in tumor suppression pathways and cell cycle regulation [25]. In 2010, the intergenic lncRNA (lincRNA)-p21Cip1/WAF1 (also known as CDKN1A) was shown to modulate the p53-dependent gene expression network in cancer [26]. In 2011, Gibb et al. analyzed 272 human Serial Analysis of Gene Expression (SAGE) libraries to delineate lncRNA expression patterns across 26 normal tissues and 19 cancers, revealing extensive tissue-specific lncRNA expression and highly aberrant lncRNA profiles in tumors. Dysregulated lncRNAs, including MEG3, HOTAIR, and MALAT1, have been identified as potential tumor suppressors or metastasis promoters, suggesting their utility as cancer biomarkers [27]. In 2012, a novel BRAF-regulated lncRNA, BRAF-activated non-coding RNA (BANCR), was identified as recurrently overexpressed in BRAF^V600E^-mutant melanomas, promoting melanoma cell migration via CXCL11 chemokine regulation [28]. Subsequent studies in 2013 revealed its tumor-suppressive role in renal cell carcinoma (RCC) by inhibiting proliferation, inducing apoptosis, and suppressing metastasis [29]. In 2019, the SNHG family of lncRNAs was found to be dysregulated in breast cancer, promoting tumor progression through miRNA sponging [30]. Moreover, in 2022, lncRNA DLEU1 was shown to regulate immune evasion and programmed cell death (particularly necroptosis) in the tumor microenvironment, influencing cancer progression [31]. A later study in 2024 identified lncRNA ZNF667-AS1 as a tumor suppressor in ovarian cancer (OC), demonstrating its underexpression in OC tissues and cell lines and its association with improved patient prognosis via TNF signaling pathway modulation [32]. In 2025, N^6^-methyladenosine (m^6^A), the most prevalent internal modification in eukaryotic mRNAs and ncRNAs, plays critical roles in RNA metabolism and cancer progression [33]. m^6^A modification alters RNA stability, localization, translation efficiency, and protein binding, thereby influencing the expression and function of oncogenic and tumor-suppressive ncRNAs [34]. Accordingly, research into the physiological and pathological roles of lncRNAs has become a major focus in this field, with potential applications as prognostic cancer biomarkers and novel therapeutic targets. 

Although there are many reviews that have listed the growing list of ncRNA functions in cancer, there are several important gaps in the literature. First, most current reviews are descriptive and list ncRNA targets and downstream effects without incorporating multi-omics data that may uncover higher order regulatory networks. Second, few efforts have been made to systematically quantify the translational landscape of ncRNA therapeutics, including a thorough evaluation of clinical trial results, delivery issues, and biomarker development. Third, most published reviews are not critical and do not differentiate between well-established mechanisms and emerging hypotheses with limited experimental support. To overcome these limitations, the present review has three distinguishing features: (1) Integration of multi-omics perspectives—We synthesize evidence from transcriptomics, epigenomics, proteomics, and metabolomics to build a systems level understanding of ncRNA regulatory networks in cancer. (2) Quantitative assessment of translational potential—We have performed a systematic review of 47 clinical trials and analyzed therapeutic modalities, delivery strategies and clinical outcomes objectively to assess the current situation of ncRNA therapeutics. (3) Critical and balanced analysis—We clearly differentiate between known mechanisms with strong evidence and new ideas that need to be substantiated throughout each section, giving readers a balanced and evidence-based view. This review takes a comprehensive and critical view to provide a reference and a forward-looking framework for the ncRNA oncology field for researchers and clinicians (Table 1).

## 2. Classification and Biogenesis of ncRNAs

**Figure 2:**
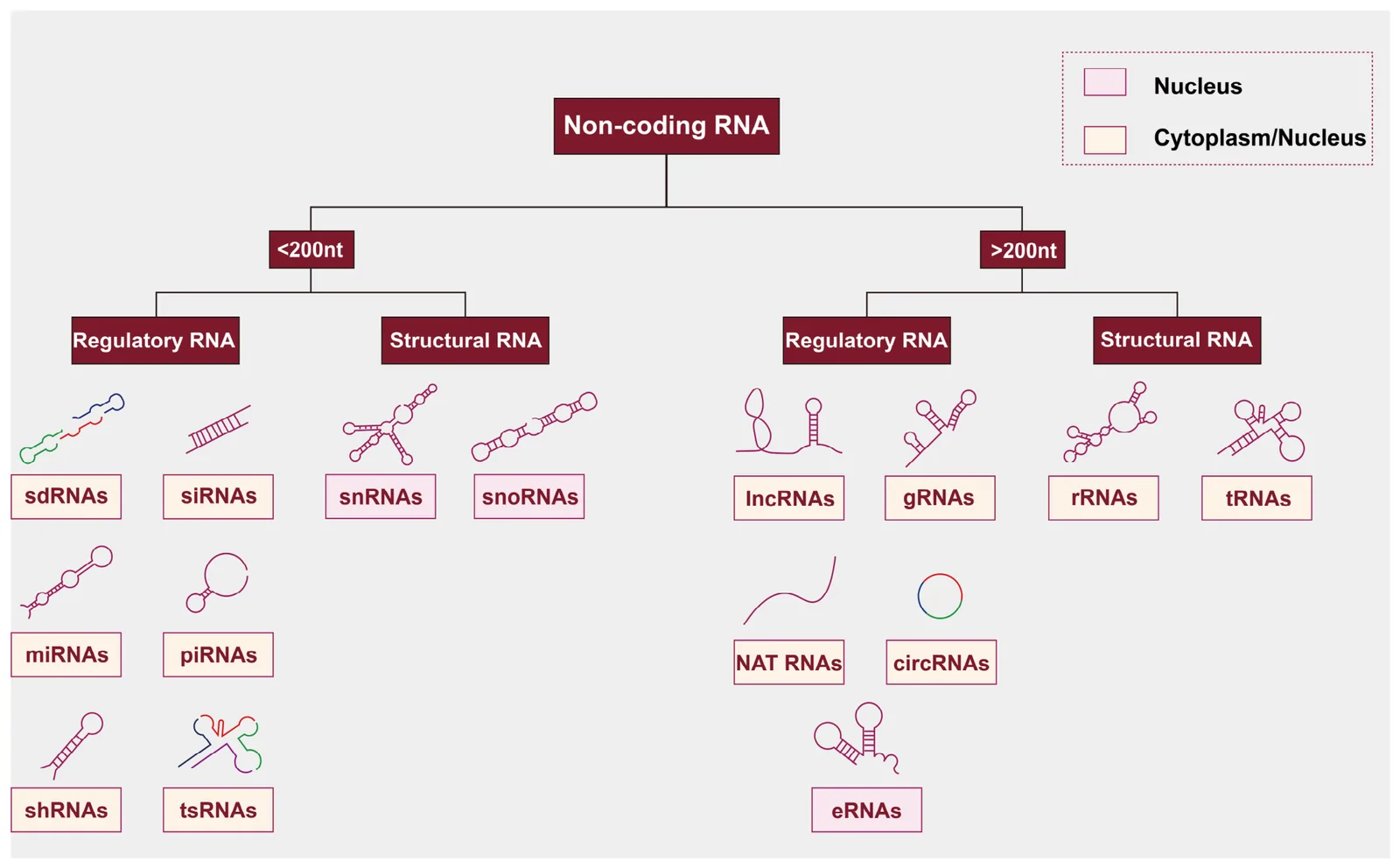
Classification of non-coding RNAs (ncRNAs). ncRNAs are classified according to their size (small (<200 nt) and long (>200 nt)), structure (linear vs. circular) and genomic origin. Functional categories and representative members are given for each class. Bottom panel compares the key features of the three main categories: small ncRNAs (linear, 18–200 nt, moderate stability, high conservation, post-transcriptional silencing); lncRNAs (linear spliced, >200 nt, low stability, low-moderate conservation, epigenetic/transcriptional control); circRNAs (covalently closed loop, variable size, high stability, moderate conservation, miRNA sponge and RBP binding). The biogenesis of circRNAs through back-splicing and their resistance to exonuclease-mediated degradation make them an independent topological class of ncRNAs. Structural ncRNAs (rRNA, tRNA, snRNA, snoRNA) and regulatory ncRNAs (miRNA, siRNA, piRNA, lncRNA, circRNA) are distinguished.

The classification of ncRNAs is based on their length, function, and genomic location. This section provides a systematic overview of the classification of ncRNAs and their biological significance. The following outlines the classification of ncRNAs (Figure 2).

### 2.1. LncRNAs

**Figure 3:**
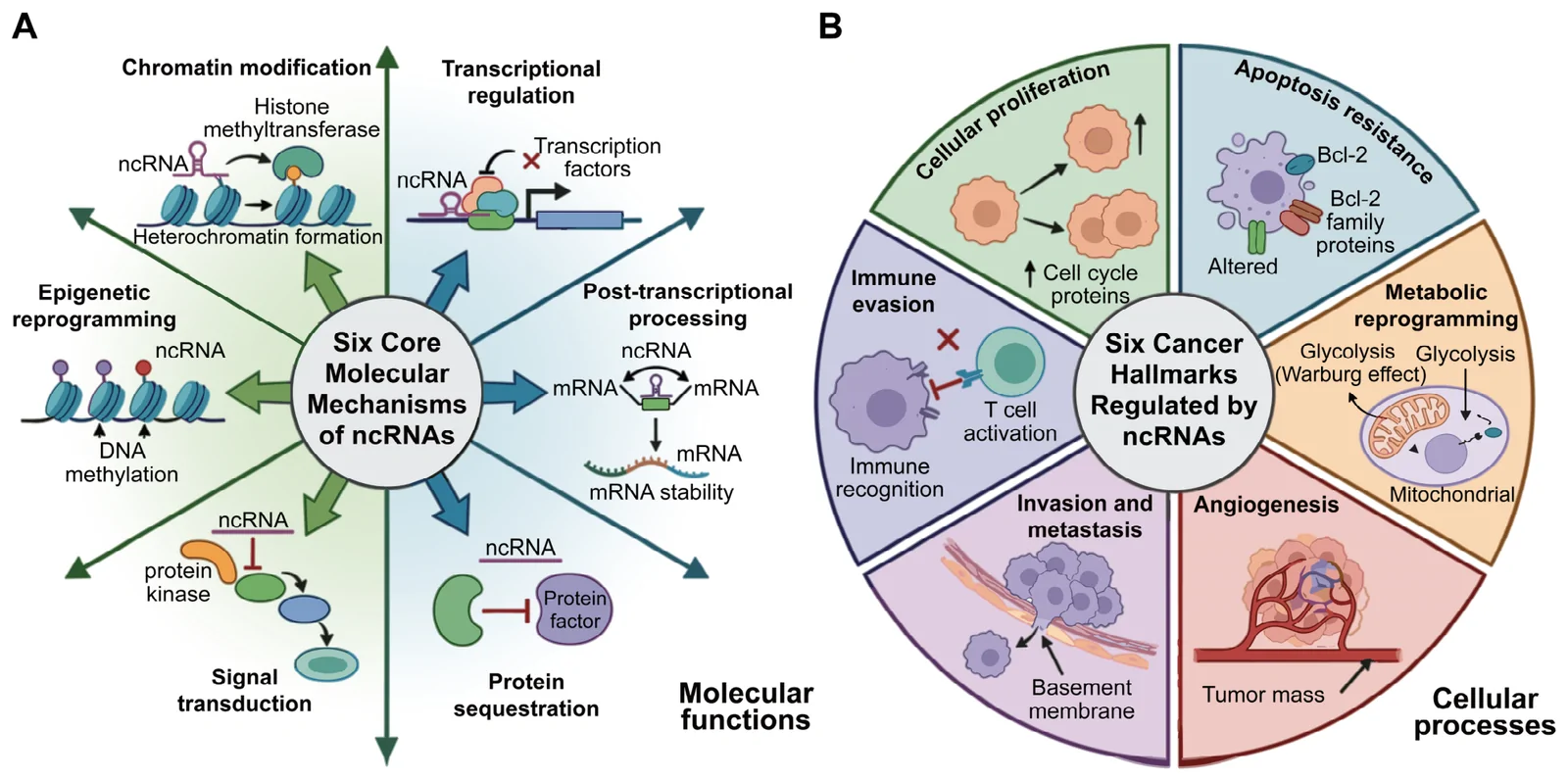
Functional classification of ncRNAs in cancer. (**A**) Molecular functions: The central diagram depicts six core molecular mechanisms through which ncRNAs operate in cancer cells, including chromatin modification, transcriptional regulation, post-transcriptional processing, protein sequestration, signal transduction, and epigenetic reprogramming. (**B**) Cellular processes: The diagram illustrates six cancer hallmarks regulated by ncRNAs, encompassing cellular proliferation, apoptosis resistance, metabolic reprogramming, angiogenesis, invasion and metastasis, and immune evasion.

Long non-coding RNAs (lncRNAs) are transcripts longer than 200 nt in length that do not produce functional proteins. Most of the lncRNAs are not protein coding, but a small fraction of them are known to encode functional peptides [35]. In addition to lncRNAs, some circRNAs have been shown to act as templates for protein translation [36], which further broadens the functional spectrum of the non-coding transcriptome. They are known to be low in both sequence conservation and expression. Importantly, lncRNAs can be found in all subcellular compartments, such as in the nucleus, cytoplasm, or in multiple cellular foci [37]. These molecules have a variety of regulatory activities in vivo including modulation of protein modifications, chromatin remodeling, protein activity and RNA metabolism [38]. Most lncRNAs are polyadenylated and spliced from multiple exons, like mRNAs [35]. They are transcribed by RNA polymerase II and their transcription is initiated by trimethylation of lysine 4 of histone H3 (H3K4me3) [39]. lncRNAs are also synthesized in a similar manner to mRNAs but do not get translated into proteins. In animals, there is currently evidence that lncRNAs are involved in many biological processes such as gene expression, genetic imprinting, dosage compensation, chromatin modification, apoptosis regulation, alternative splicing, cell differentiation and cell cycle [40]. Moreover, they have important roles in the initiation of diseases and in the process of carcinogenesis (Figure 3).

The characteristics of lncRNAs are determined by their diverse types and origins. lncRNAs can be classified into lincRNAs, intron-encoded transcripts (incRNAs), and natural antisense transcripts (NATs), which can be further subdivided based on their processing mechanisms [41]. Conventional linear lncRNAs include lincRNAs, incRNAs, and NATs [42]. Distinct from linear lncRNAs, circRNAs represent a separate topological class of ncRNAs characterized by covalently closed loop structures without 5′ caps or 3′ poly(A) tails (Section 2.3). While some circRNAs exceed 200 nt in length, their biogenesis via back-splicing and their resistance to exonuclease-mediated degradation distinguish them from linear lncRNAs. Therefore, in this review, circRNAs are treated as an independent category parallel to linear lncRNAs and small ncRNAs (Figure 2) [15]. Each type of lncRNA is produced via specific mechanisms and exhibits distinct regulatory properties in cis or trans [35]. Most linear lncRNAs are transcribed by RNA polymerase II (Pol II), exhibit lower conservation compared to mRNAs, have lower abundance, high tissue-specific expression, and inefficient splicing [39]. Most circRNAs are generated through exon circularization during mRNA splicing and are exported to the cytoplasm [15]. 

### 2.2. MicroRNAs

MicroRNAs represent an important class of endogenous regulatory ncRNAs in eukaryotes [43]. They are small, single-stranded, ncRNAs with lengths ranging from 18 to 25 nucleotides [44]. In cells, miRNAs are initially transcribed by RNA polymerase II into primary miRNAs (pri-miRNAs), which are then processed and transported to the cytoplasm to form precursor miRNAs (pre-miRNAs) with a characteristic stem-loop structure. These pre-miRNAs are further processed in the cytoplasm to generate mature miRNAs. Mature miRNAs function by binding to the 3′ untranslated region (UTR) of target mRNAs via base-pairing (either complete or incomplete), inhibiting translation and thereby silencing gene expression [45]. Extensive evidence confirms the critical role of miRNAs in biological processes and their association with human diseases due to abnormal miRNA regulation [46]. Moreover, miRNAs regulate protein synthesis by promoting mRNA degradation, inhibiting translation initiation, or participating in transcriptional and post-transcriptional regulation [47]. Changes in miRNA expression can alter the gene expression profiles involved in various biological processes, thereby contributing to the development of numerous human diseases [46]. Due to their high stability in bodily fluids, circulating miRNAs are considered promising biomarkers for tumor diagnosis and prognosis [45].

**Figure 4:**
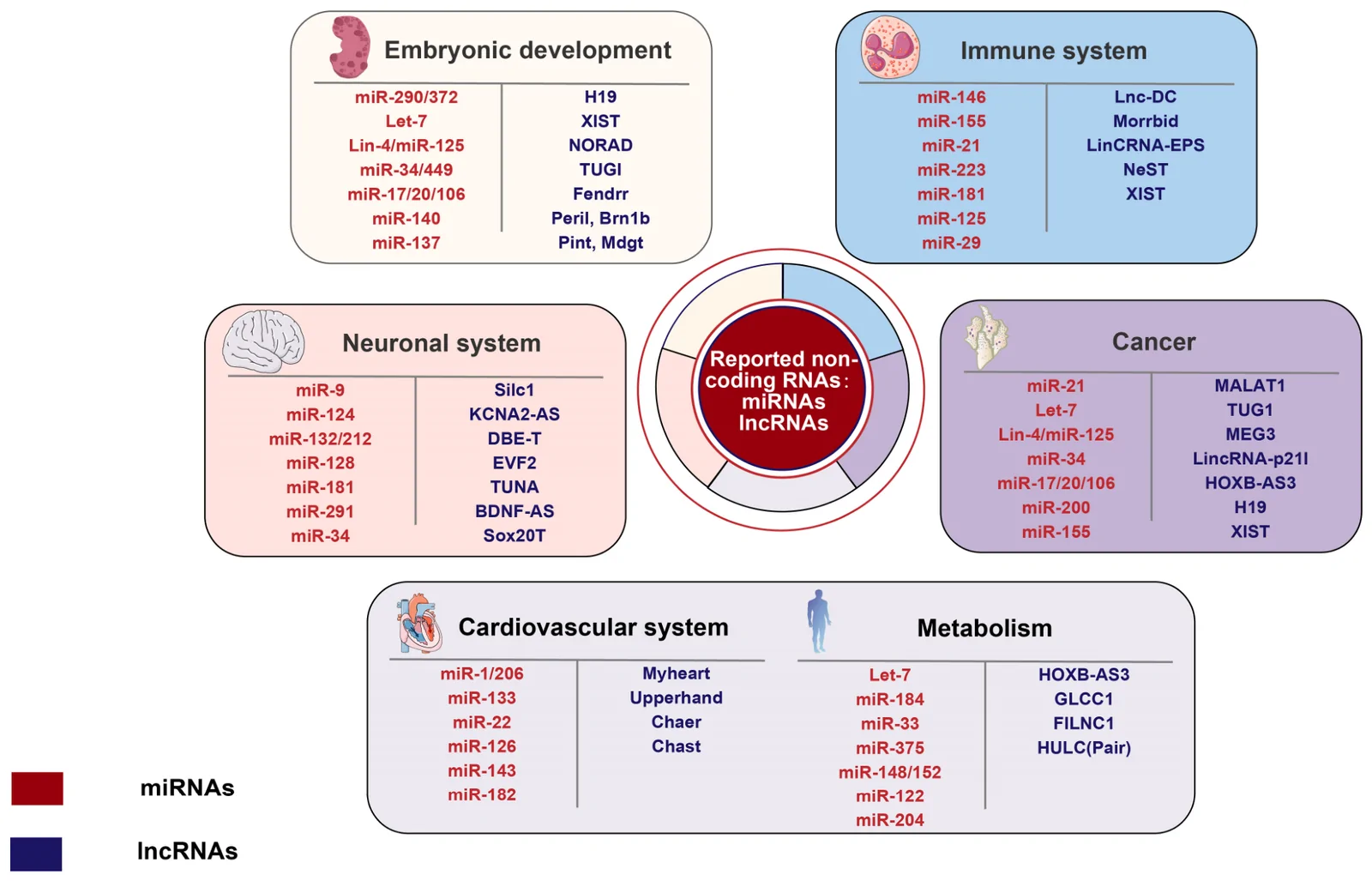
Representative ncRNAs with validated roles in cancer. The table columns are: ncRNA name, type (miRNA/lncRNA/circRNA), primary cancer types, key molecular mechanism, and functional classification. Oncogenic (red), tumor-suppressive (blue), context-dependent (purple). Criteria for selection: (1) validated by at least three independent studies from different research groups; (2) mechanistic studies that show the proteins are functional; (3) therapeutic relevance based on preclinical or clinical evidence. Included ncRNAs: HOTAIR, MALAT1, ANRIL, MEG3, H19, GAS5 (lncRNAs); miR-21, miR-34a, let-7, miR-155, miR-29b, miR-200c (miRNAs); ciRS-7, circHIPK3, circFOXO3, circCCDC66 (circRNAs).

Importantly, miRNAs typically exhibit tissue-specific, temporal, and spatial expression patterns, enabling precise regulation of gene expression. In 1993, Lee et al. discovered the first miRNA, named lin-4, in the nematode *Caenorhabditis elegans* (*C. elegans*). Lin-4 was found to incompletely base-pair with the 3′ UTR of lin-14 mRNA, inhibiting lin-14 expression and controlling larval transition from stage L1 to stage L2 in *C. elegans* [48]. In 2000, Reinhart et al. identified miRNA-let-7 and its target gene lin-41, also in *C. elegans*. Both of these early discoveries involve miRNAs binding with partial complementarity to the 3’ UTR of their target mRNAs to inhibit translation and influence *C. elegans* development [49]. Following these initial findings, more miRNAs were discovered. In 2001, a report in Science reported the identification of nearly 100 miRNAs in nematodes and humans by researchers in the United States and Germany [50]. The functions of these newly discovered miRNAs became a significant focus of study. In 2002, Calin et al. discovered that mir-15a and mir-16-1, an miRNA cluster located at chromosome region 13q14.3, are frequently deleted in chronic lymphocytic leukemia [51]. Their expression levels were significantly reduced in cancer samples, while their target gene BCL-2, which has anti-apoptotic functions, was significantly upregulated, promoting cancer cell growth and contributing to carcinogenesis [52] (Figure 4). Despite the identification of numerous miRNAs, the functions of many miRNAs in tumors remain unclear. 

### 2.3. Circular RNAs

CircRNAs represent a class of non-coding RNA molecules characterized by a closed-loop structure without 5′ caps or 3′ poly(A) tails [15]. This unique loop structure confers circRNAs with high stability and resistance to nuclease degradation, enabling them to persist in cells for extended periods [53]. Their length varies widely, ranging from dozens to thousands of nucleotides [54]. Based on their origins and functions, circRNAs can be classified into exon-derived circular RNAs (ecircRNAs), intron-derived circular RNAs (ciRNAs), and exon–intron hybrid circular RNAs (EIciRNAs) [55]. Compared to linear RNAs, circRNAs exhibit greater resistance to exonucleases and ribonucleases [53]. In addition, circRNAs exhibit structural conservation, stable sequences, and tissue-specific expression patterns [55]. With the progress of high-throughput sequencing and bioinformatics technologies, an increasing number of circRNAs have been identified. It is now understood that circRNAs can function as “sponges” for miRNAs to regulate their activity, bind to RNA-binding proteins (RBPs) to modulate their function and localization, and in some cases, regulate transcription or encode proteins [56]. Furthermore, pseudogenes derived from circRNAs may integrate into the genome.

The biogenesis of circRNAs primarily occurs through the reverse splicing of RNA precursors. Currently, the following mechanisms are understood to be involved in circRNA biogenesis: (1) Lasso-driven circularization: During the canonical process of splicing, intronic lassos are typically degraded; however, in some instances, specific splicing signals can connect the two ends of the intronic lasso, forming a circular structure and generating ciRNAs [57]. (2) Exon skipping and reverse splicing: During gene transcription, some exons may be skipped, while adjacent exons can be joined together via reverse splicing, leading to the formation of ecircRNAs. This reverse splicing process may be regulated by various factors, such as RBPs and splicing factors [58]. (3) Complementary pairing of flanking introns: Complementary base-pairing regions within intron sequences flanking exons can bring exons closer together and facilitate their connection through base-pairing, thereby promoting circRNA formation [59].

### 2.4. Small Interfering RNAs (siRNAs)

It is now understood that siRNAs are double-stranded RNA molecules, typically 21–23 nucleotides in length [60]. These molecules form a double-helix structure through base-pairing complementarity. One strand, the guide strand, binds to the RNA-induced silencing complex (RISC), while the other strand, the passenger strand, is degraded [61]. The 5′ end of the guide strand usually carries a phosphate group, and its 3′ end protrudes by 2–3 nucleotides. This structural feature is critical for the binding and activation of siRNAs with RISC [62]. siRNAs primarily function via the RNA interference (RNAi) pathway. When double-stranded RNA, whether exogenous or endogenous, is present in the cell, it is cleaved by the ribonuclease Dicer into siRNAs [63]. Subsequently, the siRNAs bind to the core protein Argonaute (Ago) within RISC, forming the active RISC [60]. Within this complex, the guide strand recognizes and binds to target mRNA complementary to its sequence through base-pairing complementarity. The Ago protein, which exhibits endonuclease activity, then cleaves the target mRNA at a specific site, leading to its degradation and thereby silencing the expression of the corresponding gene [64].

Through specific siRNA design, specific knockdown of gene expression can be achieved at the cellular level and the effects of these siRNAs on the behaviors of tumor cells, including proliferation, apoptosis, migration and invasion, can be investigated, providing deeper insights into the roles of tumor-related genes in tumorigenesis and progression [60]. The proto-oncogene KRAS has been shown to be essential for the development of several types of tumors and its knockdown has been shown to have a significant impact on the proliferation and migration of tumor cells using siRNAs targeting KRAS [65]. The formation of tumors can be a complex process with many signaling pathways involved. Importantly, siRNAs can be used to investigate the role and interaction of important molecules in these pathways. For example, the researchers have identified that the abnormal activation of this pathway is closely related to the survival, proliferation and metabolism of tumor cells by using siRNAs to knock down various components of the PI3K/AKT/mTOR signaling pathway, providing valuable clues for understanding tumor pathogenesis [66].

## 3. Mechanisms of Non-Coding RNAs in Cancer

### 3.1. ncRNA-Mediated Epigenetic Regulation in Cancer

ncRNAs regulate epigenetic landscapes through multiple mechanisms, including the recruitment of chromatin-modifying complexes, modulation of DNA methylation machinery, and regulation of histone modification patterns [67]. Established mechanisms—supported by extensive experimental evidence including ChIP-seq, RNA-seq, and functional rescue studies—include lncRNA-guided recruitment of PRC2 (HOTAIR, ANRIL) and miRNA-mediated targeting of DNMTs (miR-29 family) [22]. Emerging hypotheses—requiring further validation through genetic models and clinical correlation—include circRNA-mediated regulation of chromatin accessibility and the proposed role of ncRNAs in three-dimensional genome organization [14]. 

#### 3.1.1. Regulatory Mechanisms of ncRNAs in Cancer

**Figure 5:**
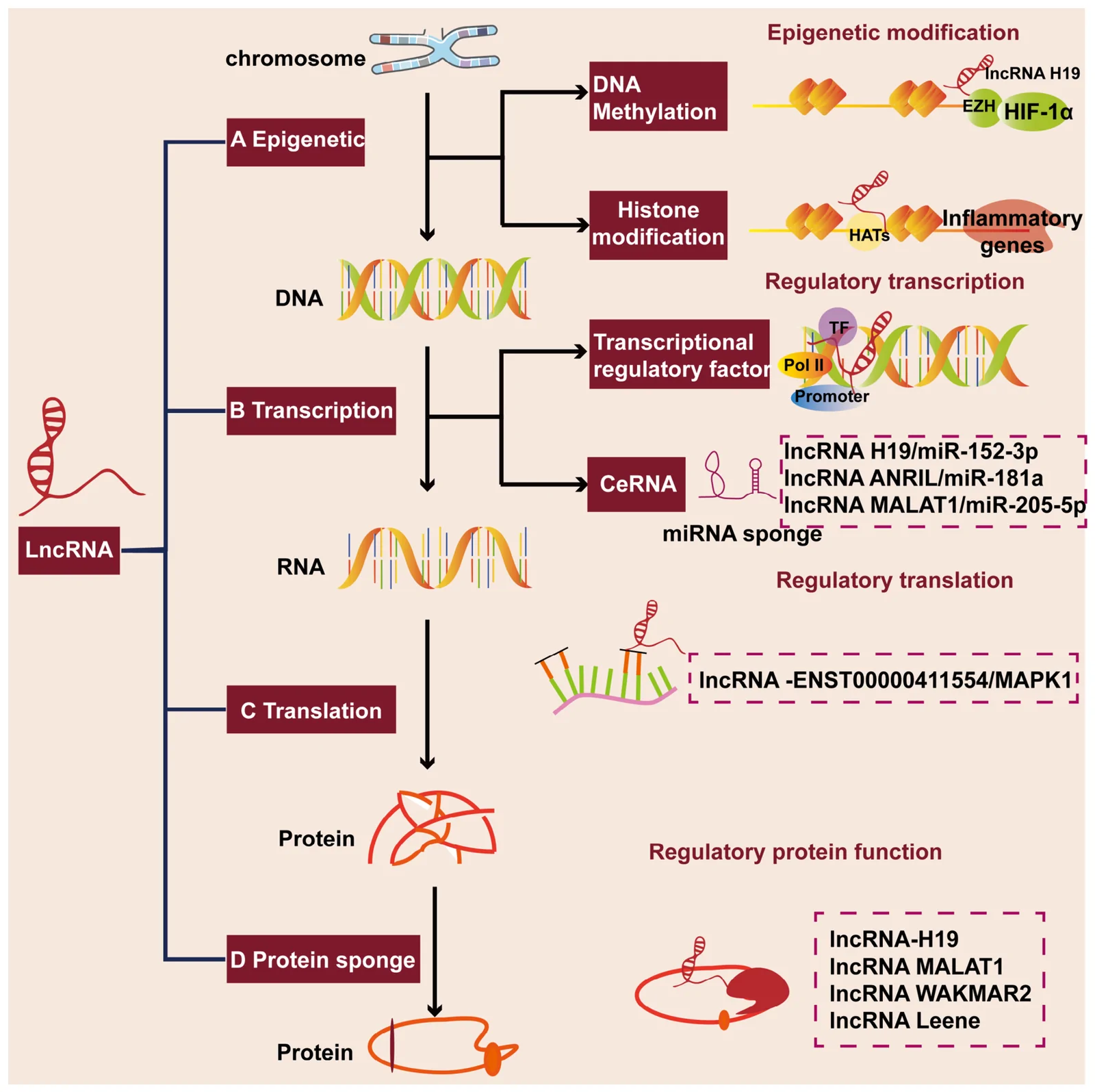
Epigenetic modifications regulated by ncRNAs in cancer. DNA methylation, showing ncRNA-mediated regulation of DNA methyltransferases (DNMTs) and demethylases (TETs). Histone modification, depicting ncRNA-guided recruitment of histone-modifying complexes including PRC1, PRC2, and histone acetyltransferases/deacetylases (HATs/HDACs). Representative miRNAs and lncRNAs are indicated for each modification type.

ncRNAs are involved in the development of cancer in four major molecular mechanisms (Figure 5). First, chromatin remodeling: lncRNAs like HOTAIR and ANRIL bind to chromatin-modifying complexes to turn off tumor suppressor genes or on oncogenes. Second, transcriptional regulation: ncRNAs regulate transcription through interaction with transcription factors and RNA polymerase II [67]. Third, post-transcriptional processing: miRNAs interact with the 3′ UTR of target mRNAs to inhibit translation or degrade the mRNA. Fourth, signal transduction: ncRNAs act as molecular scaffolds for complex assembly of signaling [67,68]. Each mechanism is explained in detail in subsequent sections and examples are chosen that have been well experimentally validated and are clinically relevant.

#### 3.1.2. Regulatory Mechanism of miRNAs in Chromatin Remodeling

miRNAs regulate chromatin remodeling by targeting key chromatin-modifying enzymes and transcription factors [68,69]. For example, miR-29 targets DNA methyltransferases DNMT3A and DNMT3B, leading to genome-wide alterations in DNA methylation patterns, while miR-101 represses EZH2 expression, thereby modulating H3K27me3 levels at target gene promoters [70]. These findings establish miRNAs as upstream regulators of the epigenetic machinery in cancer cells.

Regulation of histone modification enzymes by miRNAs. miRNAs can modify the chromatin structure by directly binding to histone methyltransferases (HMTs) or histone deacetylases (HDACs) [71]. For instance, miR-26a and miR-101 can inhibit the catalytic subunit of PRC2, EZH2, which decreases the amount of H3K27me3, thereby relieving transcriptional silencing [72]. The miR-128 family targets SUZ12 and BMI1, which in turn reverse chromatin condensation and activate tumor suppressor genes like PTEN [73]. The miR-34a and miR-449a each bind to HDACs, leading to hyperacetylation of H3K9/K27 and chromatin opening, respectively [74]. 

Indirect chromatin regulation via transcription factors. miRNAs can also indirectly regulate the recruitment of chromatin remodeling complexes by targeting transcription factors [71]. For example, the miR-200 family upregulates E-cadherin by downregulating ZEB1/ZEB2 which inhibits chromatin remodeling associated with EMT [75]. In KIT mutant leukemia, miR-29b is suppressed by MYC, which results in an increase in the expression of Sp1 and recruitment of SWI/SNF to the KIT promoter [76]. 

Crosstalk between miRNA and lncRNA in chromatin regulation. lncRNAs like HOTAIR act as scaffolds for chromatin-modifying complexes (PRC2 at 5′ end, LSD1/CoREST at 3′ end) and silence target genes such as CDKN2A [77]. This process is counteracted by miR-7, miR-206 and miR-146a-5p, which inhibit the expression of HOTAIR, impair PRC2 genomic localization, and reverse metastasis-associated chromatin remodeling, respectively [78]. 

DNA methylation-miRNA feedback loops. miRNAs control the balance between DNA methylation and histone modification by targeting DNMTs [79]. For instance, miR-101 has been shown to inhibit DNMT3A and thereby hypermethylate CpG islands at the promoters of tumor suppressor genes, which leads to gene activation [80]. In prostate cancer, on the other hand, DNMT1 hypermethylates miR-26a, which results in overexpression of LSD1, H3K4 demethylation, and chromatin compaction [81].

### 3.2. Transcriptional and Post-Transcriptional Control

#### 3.2.1. miRNA-Mediated Regulation of Oncogenes and Tumor Suppressors

In the complex network of gene expression regulation, the interplay between both transcriptional and post-transcriptional regulation determines cell fate and functional phenotypes by dynamically controlling mRNA synthesis, stability, and translation efficiency. As a core member of the non-coding RNA family, miRNAs play a complex dual regulatory role in tumor development by targeting oncogenes such as MYC and KRAS or tumor suppressor genes such as PTEN and TP53, thereby driving malignant transformation or inhibiting tumor progression [47]. In this section, we systematically review recent advances in miRNA-mediated tumor regulation from the perspectives of molecular mechanisms and functional studies. 

The role of transcriptional condensates in cancer: Gene transcription is spatiotemporally regulated by dynamic assembly of transcriptional condensates via liquid–liquid phase separation (LLPS). These condensates form microenvironments with high concentrations of RNA polymerase II and coactivators that can regulate gene expression [81]. In AML, abnormal stabilization of transcriptional condensates leads to the self-renewal of leukemia stem cells by the aberrant activation of the HOXA gene cluster by the oncoprotein NUP98-HOXA9 [82]. This mechanism shows the role of condensate dysregulation in the initiation of oncogenic transcription programs. These processes are further fine-tuned by post-transcriptional regulation of mRNA stability and translation efficiency by miRNAs [83]. Bidirectional regulatory networks of miRNAs in tumors: Specific miRNAs can drive the malignant phenotype of tumors by targeting oncogenes or activating oncogenic pathways. For instance, miR-21 directly represses PTEN (a negative regulator of the PI3K/AKT pathway) and PDCD4 (an apoptosis-promoting factor), promoting cell proliferation and invasion [84]. High miR-21 expression has been reported to be significantly correlated with 5-fluorouracil resistance in colorectal cancer [85]. The miR-221/222 family contributes to drug resistance in breast cancer by targeting p27^Kip1 (a cell cycle inhibitor) and ERα (estrogen receptor alpha), promoting G1/S phase transition and inducing tamoxifen resistance [86]. Single-cell sequencing has revealed that miR-221 is specifically enriched in breast cancer stem cells. Current evidence suggests that ome miRNAs exert anti-tumor effects by suppressing oncogenes or restoring tumor-suppressive pathways. The miR-15a/16-1 cluster regulates leukemia; in chronic lymphocytic leukemia (CLL), chromosome 13q14 deletion leads to loss of this cluster, deregulating BCL2 (an anti-apoptotic gene) and promoting cell survival [87]. Clinical cohort studies have shown a 40% reduction in overall survival (OS) in patients with low miR-15a expression [88]. The let-7 family targets oncogenes such as RAS (MAPK pathway) and HMGA2 (chromatin remodeling factor), blocking lung cancer cell proliferation. Single-cell RNA sequencing indicates that low let-7 expression is correlated with enhanced cancer stem cell properties [89]. 

Feedback loops and epigenetic regulation: miRNAs and epigenetic modifications form bidirectional regulatory networks that amplify the malignant phenotype of tumors. For example, in chronic myeloid leukemia (CML), ABL1 kinase activity induces hypermethylation of the miR-203 promoter via DNMT1, silencing miR-203 and deregulating ABL1 inhibition, creating a pro-oncogenic feedback loop [90]. The miR-148a/DNMT1 axis reduces promoter methylation levels of tumor suppressor genes such as CDKN2B by targeting DNMT1, restoring their transcriptional activity [91]. In hepatocellular carcinoma, miR-148a expression is positively correlated with patients’ progression-free survival (PFS) [92]. Moreover, miRNAs represent key targets for precision tumor therapy through the synergistic effects of transcriptional and post-transcriptional regulatory networks. Future studies should integrate single-cell multi-omics, CRISPR screening, and artificial intelligence models to resolve the dynamics and heterogeneity of miRNA–target gene interactions and optimize delivery systems for clinical translation. Indeed, addressing these challenges will facilitate the transition of miRNA-based individualized therapies from the laboratory to clinical practice.

#### 3.2.2. CircRNAs Sponging miRNAs or RNA-Binding Proteins

circRNAs can act as competing endogenous RNAs (ceRNAs) by binding to miRNAs (the “sponge effect”) or to RNA-binding proteins (RBPs) [93]. In this section, the bi-axial regulatory network of circRNAs with miRNAs and RBPs in cancers is reviewed, particularly focusing on examples that have been mechanistically validated. Oncogenic signaling of circRNA-miRNA sponging. circRNAs bind miRNAs by binding to miRNA response elements (MREs) and derepress target oncogenes. ciRS-7 (CDR1as) is a miR-7 binding site containing over 70 sites, which releases EGFR, RAF1 and IRS2, leading to the activation of PI3K/AKT and MAPK in glioblastoma [94]. circHIPK3 sequesters miR-124 and miR-29b which are involved in autophagy and cell cycle progression, respectively [95]. circCCDC66 can simultaneously bind to miR-33b and miR-93, as well as synergistically activate the Wnt/β-catenin pathway in CRC [96]. Interestingly, several circRNAs can target the same miRNA: circPVT1 and circHIPK3 bind to miR-21, leading to chemoresistance [97].

The role of circRNA-RBP interaction in cell cycle and transcriptional regulation. circRNAs interact with RBPs to regulate their localization, stability and activity in the cell [98]. circFoxo3 binds to CDK2 and p21Cip1 to block cell cycle progression [99], and circANRIL binds to PES1 to cause nucleolar stress and p53 activation [100]. Nuclear-localized circRNAs like EIciRNA-EIF3J bind with U1 snRNP and RNA polymerase II to facilitate elongation of transcription [101]. In breast cancer, circMYO10 can simultaneously bind to miR-370-3p to derepress PTEN and to bind to IGF2BP3 to enhance MYC mRNA stability, synergistically supporting metastasis [95] (Table 2).

circRNAs were selected for inclusion based on the following criteria: (1) mechanistic studies demonstrating direct regulation of a defined signaling pathway component; (2) functional validation showing altered pathway activity upon circRNA modulation; (3) cancer relevance supported by in vivo or clinical evidence; (4) minimum two independent publications confirming the regulatory interaction [14].

### 3.3. Signal Transduction and Tumor Microenvironment

#### 3.3.1. ncRNAs Regulating Wnt/β-Catenin, PI3K/AKT, and NF-κB Pathways

Tumorigenesis is a multi-stage and multi-gene regulated process, in which the signal transduction pathways play a key role. Wnt/β-catenin, PI3K/AKT and NF-κB pathways play important roles in cell proliferation, survival, migration and immune regulation, respectively [102]. Moreover, the complexity and dynamic changes of the tumor microenvironment (TME), as an essential matrix for tumor growth and metastasis, should not be overlooked in tumorigenesis. Over the past few years, many studies have shown that different categories of ncRNAs directly or indirectly have an impact on the structure and functioning of the TME [103]. Thus, the study of the regulatory function of ncRNAs in these important pathways may shed light on the molecular mechanism of tumors and offer a fresh perspective for accurate diagnosis and treatment.

- ncRNAs regulate the Wnt/β-catenin pathway

The Wnt/β-catenin pathway regulates cell proliferation and survival, and abnormal activation of this pathway causes the accumulation of β-catenin in the nucleus and the induction of target genes, which leads to tumorigenesis [104]. This pathway is directly regulated by miRNAs, including miR-34 which targets β-catenin, and the miR-200 family which targets Frizzled receptors (FZDs); other miRNAs target upstream regulators including DKK1 and SFRP members [68,105,106]. lncRNAs and circRNAs indirectly promote Wnt/β-catenin signaling as ceRNAs that sequester these negative regulators. For instance, lncRNA CCAT2 and ciRS-7 sponge miRNAs that are normally involved in inhibiting the expression of β-catenin, which is essential for tumor progression [94,107]. Some lncRNAs also bind directly to chromatin modification complexes to regulate the transcription of Wnt pathway genes [108]. In addition to the tumor cell-intrinsic effects, Wnt signaling in cancer-associated fibroblasts (CAFs) has tumor microenvironment remodeling functions by stimulating cytokine secretion [109].

b. Regulation of the PI3K/AKT pathway by ncRNAs

The PI3K/AKT pathway is often hyperactivated in cancers, promoting survival, proliferation and resistance to therapy [110]. miR-126 and miR-145 directly target subunits of the PI3K pathway or AKT to inhibit signaling, while oncogenic miR-21 activates signaling by targeting PTEN [111]. In many cancers, the upregulation of miR-21 is often associated with chemoresistance. This axis is also regulated by lncRNAs and circRNAs by ceRNA mechanisms, where HOTAIR and circRNA_001569 sequester miR-21 or miR-126, respectively, leading to the derepression of PTEN and suppression of pathway activity [112,113]. Other ncRNAs also directly bind to components of the PI3K/AKT signaling complex, which affects the propagation of downstream signals [114]. In this way, ncRNAs can alter the tumor microenvironment by controlling the secretion of cytokines and the induction of angiogenesis [115].

c. Regulation of the NF-κB pathway by ncRNAs

By promoting survival and immune evasion, aberrant activation of NF-κB is a driver of inflammation-associated cancers [116]. miR-146a and miR-155 regulate the activity of upstream activators IRAK1 and TRAF6, respectively [117]. miR-146a is involved in negative feedback: miR-146a is induced by NF-κB and in turn inhibits inflammatory responses [118]. lncRNA NKILA directly binds to the NF-κB complex, preventing its translocation to the nucleus and thus blocking downstream inflammatory gene expression [119]. The role of circRNAs in fine tuning the intracellular inflammatory signaling is further regulated by the ability of circRNAs to sponge miR-155 or miR-146a [120].

Together, ncRNAs control the activity of the Wnt/β-catenin, PI3K/AKT, and NF-κB pathways at multiple levels, impacting cytokine production, immune cell function, and stromal function in the TME [121,122]. This pathway crosstalk is a key factor in tumor progression, therapeutic resistance, and the immunosuppressive tumor microenvironment. In the future, context-specific mapping of ncRNA–pathway interactions in different cancer types should be prioritized to facilitate precision therapeutic strategies. 

#### 3.3.2. Exosomal ncRNAs in Intercellular Communication

Exosomes facilitate intercellular communication by carrying intercellular ncRNAs from tumor cells to the microenvironment [123,124]. This section discusses the role of exosomal ncRNA in four major areas: signal transduction, immune regulation, metastasis, and clinical translation. ncRNAs in pathways activation in exosomes: Exosomes derived from the tumor activate the PI3K/AKT and Wnt/β-catenin signaling pathways in neighboring stromal cells, thereby inducing proliferation, EMT and metastasis [125,126]. ncRNA in immune evasion in exosome: Exosomal lncRNAs and miRNAs regulate tumor-associated macrophages (TAMs), myeloid-derived suppressor cells (MDSCs), and T cells to create an immunosuppressive tumor microenvironment, which aids in immune escape [127].

ncRNAs in metastasis in the exosome: Exosomal ncRNAs activate MMPs and trigger stromal remodeling and ECM modification, which facilitates angiogenesis [125]. They also cause EMT by introducing Snail and ZEB regulators into recipient cells, which reduces cell–cell adhesion and increases cell migration. Clinical implications: Exosomal ncRNAs are potential liquid biopsy biomarkers due to their stability in bodily fluids and their ability to reflect tumor status, and they can be used for early diagnosis and disease monitoring [67]. From a therapeutic point of view, blocking the biogenesis of exosomes or altering their ncRNA content could break the crosstalk between the tumor microenvironment and inhibit the disease progression [128]. The exosomal ncRNAs are emerging as attractive targets for early diagnosis, prognosis evaluation and targeted therapy due to their pivotal role in tumor proliferation, invasion, metastasis and immune evasion [125]. Future research needs to be directed towards elucidating the selective packaging and targeted delivery of exosomal ncRNAs and their multilevel regulatory mechanisms. The study will be highly useful in the development of more accurate and effective anti-tumor therapeutic treatmentss [128].

### 3.4. Metabolic Reprogramming

ncRNAs are able to directly target metabolic enzyme mRNAs, scaffold metabolic enzyme complexes, and modulate metabolic cofactors for epigenetic enzymes, which are not only downstream effects of oncogenic signaling but also mechanisms through which metabolic reprogramming (Warburg effect, glutamine addiction, lipid synthesis) is actively shaped [38]. These mechanisms are explored in this section, and it is understood that metabolic regulation is one of the mechanisms of action of ncRNAs, as well as apoptosis, autophagy and cell cycle control (Section 3.5).

#### 3.4.1. From Molecular to Precise Regulation of Metabolic Reprogramming: The ncRNA Regulatory Network of Glycolysis

Glycolysis is one of the most important pathways in the metabolic rewiring of the tumor, providing energy and biosynthetic precursors to cancer cells via increased glucose uptake, upregulation of rate-limiting enzymes, and increased lactate production [129]. ncRNAs are involved in the regulation of the glycolytic flux in a multilevel manner by targeting key glycolytic components including glucose transporters, hexokinase, phosphofructokinase, and lactate dehydrogenase. This network acts as a key molecular hub that connects oncogenic signals to metabolic phenotypes.

- miRNAs as “molecular switches” for modulation of glycolysis

miRNAs have bidirectional regulation of glycolysis by targeting metabolic enzymes and their upstream regulators [130]. Oncogenic miR-21 acts in two ways to increase glycolysis: by inhibiting PTEN, which activates the transcription of GLUT1 by PI3K/AKT, and by binding to the mRNA of HK2 and increasing its stability [131,132]. In contrast, miR-34a inhibits GLUT1 and HK2 expression by inhibiting the activity of c-Myc, but hypoxia-induced HIF-1α can counteract this effect by repressing the miR-34a promoter [133,134]. In the rate-limiting enzyme step, miR-16-5p binds to PDK4 to stimulate the production of lactate, creating a regulatory axis with lncRNA MALAT1 [135,136]. The miR-122 is a suppressor of LDHA that helps to reduce lactate production, while the viral oncoproteins (such as HBV X protein) can alter the balance by reducing the tumor-suppressive miRNAs [137,138].

b. LncRNAs as “Molecular Hubs” for Glycolytic Signaling Integration

Structural interactions between lncRNAs and glycolytic enzymes control their function and localization [139]. In breast cancer, H19 forms a G-quadruplex structure with hnRNPL, which enhances the tetramerization of PKM2 over that of PKM1, thus shifting glycolytic flux towards the glycolytic pathway [140]. In gastric cancer, MALAT1 could maintain glycolysis by two pathways: blocking the function of miR-32-5p and relieving its repression on HK2, as well as binding to VDAC1 and anchoring HK1 to mitochondria [141]. *MALAT1* knockdown impairs this integration of metabolism and survival and induces apoptosis [142].

c. CircRNAs as “sponge regulators” of glycolytic gene expression

It is well-established that circRNAs, due to their stable closed-loop structures, regulate glycolytic gene expression by acting as miRNA sponges or recruiting transcription factors [59]. In pancreatic cancer, circHIPK3 functions as a competitive endogenous RNA by sponging miR-487a-3p, thereby releasing post-transcriptional inhibition of GLUT1 and HK2 and increasing glucose uptake threefold [143]. Further mechanistic studies revealed that the loop structure of circHIPK3 recruits RNA polymerase II to the GLUT1 promoter region, where it collaborates with c-Myc to enhance transcription, forming a dual regulatory mechanism of “miRNA sponge-transcriptional activation” [144]. Importantly, this structure-based regulation provides a stable molecular scaffold for efficient glycolytic gene expression. Through post-transcriptional regulation by miRNAs, structural modulation by lncRNAs, and miRNA sponge effects mediated by circRNAs, ncRNAs have established a complex and finely tuned regulatory network spanning all stages of glycolysis, from glucose uptake to enzymatic activity and lactate production [44]. These regulatory mechanisms not only meet the energetic and biosynthetic requirements of tumor cells but also help to remodel the microenvironment and mediate therapeutic resistance through metabolites like lactate [129]. Future studies should aim to decipher the interplay between ncRNA-regulated glycolysis and other metabolic pathways, such as glutamine catabolism, lipid metabolism, and investigate the spatial and temporal specificity of ncRNA regulation using single-cell metabolomics and gene-editing technologies [38]. Notably, the ncRNA-GLUT/HK/LDHA axis could serve as a potential target to overcome the drawbacks of traditional metabolic inhibitors and could be the basis for precision therapy targeting metabolic reprogramming [145].

#### 3.4.2. ncRNA Regulatory Mechanism of Glutamine Catabolism: Precise Regulation from Uptake to Metabolic Flow

As a “multi-functional nutrient” for tumor cells, glutamine catabolism not only provides α-ketoglutarate (α-KG) for the tricarboxylic acid cycle but also supplies key precursors for nucleotide synthesis, redox homeostasis, and epigenetic modifications [129]. Indeed, ncRNAs play a central role in tumor metabolic reprogramming by regulating glutamine transport, enzyme activity, and downstream metabolic flux, forming a multilevel and multi-dimensional molecular regulatory network.

- miRNAs as “Molecular gates” for glutamine uptake and enzyme splicing

miRNAs can control glutamine metabolism by targeting transporters and glutaminase (GLS) isoforms [47]. miR-15a-5p is involved in the regulation of the glutamine transporter SLC1A5 (ASCT2) in renal cancer, decreasing the intracellular levels of glutamate and triggering oxidative stress [146]. The low expression of miR-15a-5p is associated with increased glutamine metabolism signatures and reduced overall survival, confirming its prognostic value [146]. The miR-23a/b family regulates the expression of hnRNPA1, which switches the expression of GLS from kidney-specific (K-GLS) to tumor-preferred (mGLS) and increases glutamine to glutamate conversion and nucleotide precursor supply [147,148].

b. LncRNAs as “Directional regulators” of glutamine metabolic flux

lncRNAs modify the metabolic flux of glutamine in an epigenetic manner and as scaffolding molecules [149]. In hepatocellular carcinoma, GACAT3 binds to the GLS1 promoter to promote GLS1 transcription and keep α-ketoglutarate levels up, while *GACAT3* knockout leads to activation of compensatory glycolysis through HIF-1α [150]. In colorectal cancer, CCND2-AS1 acts as a metabolic scaffold to link GLS2 with PRPS1, thereby linking glutamine-derived glutamate to purine synthesis [151]. These mechanisms are examples of the ways by which lncRNAs guide metabolic precursors towards biosynthetic pathways that are needed for proliferation.

c. CircRNAs as “molecular sensors” of glutamine stress response

circRNAs regulate adaptive glutamine metabolism in response to oxidative stress and hypoxia. CircZNF609 can alleviate the repression of GLS1 by acting as a sponge for miR-145-5p and promoting the transcription of GLS1 by binding to SP1 at the promoter region of GLS1 in pancreatic ductal adenocarcinom [152,153]. This leads to the production of α-KG, which facilitates chromatin plasticity and survival through H3K27me3 demethylation by JMJD3. miRNAs, lncRNAs and circRNAs form a coordinated regulatory network that regulates glutamine catabolism, ranging from the regulation of transporters, the switching of enzyme isoforms, metabolic flux redirection, to stress adaptation [154,155,156].

#### 3.4.3. ncRNA Regulation Network of Lipid Metabolism: Multi-Dimensional Regulation from Synthesis to Homeostasis

To satisfy the needs of rapidly proliferating cells, tumor cells rewire lipid metabolic networks to increase membrane biosynthesis and the production of signaling molecules [157]. These include an increase in fatty acid synthesis, uptake, oxidation, and a breakdown in fatty acid storage. In fact, ncRNAs are central hubs in the regulation of gene expression, control lipid metabolism at multiple levels of post-transcriptional regulation, protein interaction, and subcellular localization, and are associated with metabolic rewiring and tumor malignancy [158].

- miRNAs as core post-transcriptional regulators of fatty acid metabolism

miRNAs perform bidirectional regulation of lipid metabolism by targeting key metabolic genes [46]. miR-33a/b inhibit de novo fatty acid synthesis by targeting SREBP-1c and FASN, whereas MYC-driven suppression of miR-33a/b derepresses FASN to promote membrane phospholipid synthesis [159,160]. miR-122 inhibits fatty acid uptake and β-oxidation by targeting CD36 and CPT1A, favoring triglyceride storage [160]. Under stress conditions, p53-induced miR-192-5p targets SCD1, reducing monounsaturated fatty acid synthesis and modulating metabolic adaptation during nutrient deprivation [161].

b. LncRNAs as timing control hubs of lipid metabolism

lncRNAs regulate lipid metabolism through subcellular localization and protein interactions [155]. LIPCAR facilitates CD36 membrane localization in breast cancer, enhancing fatty acid uptake [162,163]. ADAMTS9-AS1 binds ABCA1 via a stem-loop structure in ovarian cancer, inhibiting cholesterol export and causing intracellular accumulation that activates mTOR-driven proliferation [164,165].

c. CircRNAs as structurally specific remodeling factors of lipid metabolism

circRNAs also regulate lipid metabolism through structure-specific mechanisms [59]. In lung cancer, circFASN competitively inhibits FASN-ACC complex formation, reducing fatty acid synthesis efficiency [166]. ncRNAs thus establish a multi-layered regulatory network in lipid metabolism: sequence-specific binding (miRNA-3′UTR), protein-mediated localization (lncRNA-CD36), and structural interference (circRNA steric hindrance) [167].

### 3.5. ncRNAs as Integrators of Cellular Homeostasis and Stress Responses

Beyond oncogenic signaling and metabolic rewiring, ncRNAs orchestrate fundamental cellular processes—including apoptosis, autophagy, cell cycle checkpoints, and DNA damage responses—that collectively determine tumor cell survival under stress.

Apoptosis and Autophagy: miR-34a induces apoptosis by targeting BCL-2 and CDK6, while simultaneously activating autophagy via ATG4B suppression. lncRNA MEG3 enhances p53-dependent apoptotic signaling by binding to MDM2 and preventing p53 degradation [168]. Conversely, lncRNA HULC and circRNA-FOXO3 inhibit apoptosis by sequestering miR-199a-5p and miR-136, respectively, thereby stabilizing anti-apoptotic MCL-1 [99]. The AMPK and mTOR signaling pathways serve as master regulators of cellular metabolism, coordinating cell growth, autophagy, and energy homeostasis in response to nutrient availability [169]. ncRNAs modulate these pathways by targeting core components, including AMPKα, mTOR, Raptor, and TSC1/2, thereby rewiring metabolic networks to support cancer cell survival and proliferation [67].

Cell Cycle and DNA Damage Response (DDR): The let-7 family suppresses cell cycle progression by targeting cyclins (CCND1, CCNA2) and CDKs (CDK6), whereas miR-221/222 promotes G_1_/S transition by inhibiting p27^Kip1^ and p57^Kip2^ [170]. lncRNA NORAD (non-coding RNA activated by DNA damage) functions as a molecular sponge for SFPQ (splicing factor proline/glutamine-rich), preventing SFPQ from aberrantly repressing DDR genes [171]. NORAD loss leads to genomic instability and sensitizes cells to ionizing radiation, highlighting ncRNAs as guardians of genome integrity.

Oxidative and Hypoxic Stress: Under hypoxia, HIF-1α induces a suite of hypoxia-responsive ncRNAs. lncRNA HIF1A-AS2 stabilizes HIF-1α mRNA by blocking ribosome-mediated decay, whereas miR-210 targets SDHD (succinate dehydrogenase complex subunit D), shifting metabolism toward glycolysis and suppressing mitochondrial ROS production [172]. circRNA-000203 sequesters miR-26b-5p, relieving its suppression of COL1A2 and enhancing cardiac fibrosis under oxidative stress [173]. These stress-responsive ncRNA circuits illustrate how tumors exploit ncRNA plasticity to adapt to hostile microenvironments.

### 3.6. The Dual Role of Non-Coding RNA in Cancer

**Figure 6:**
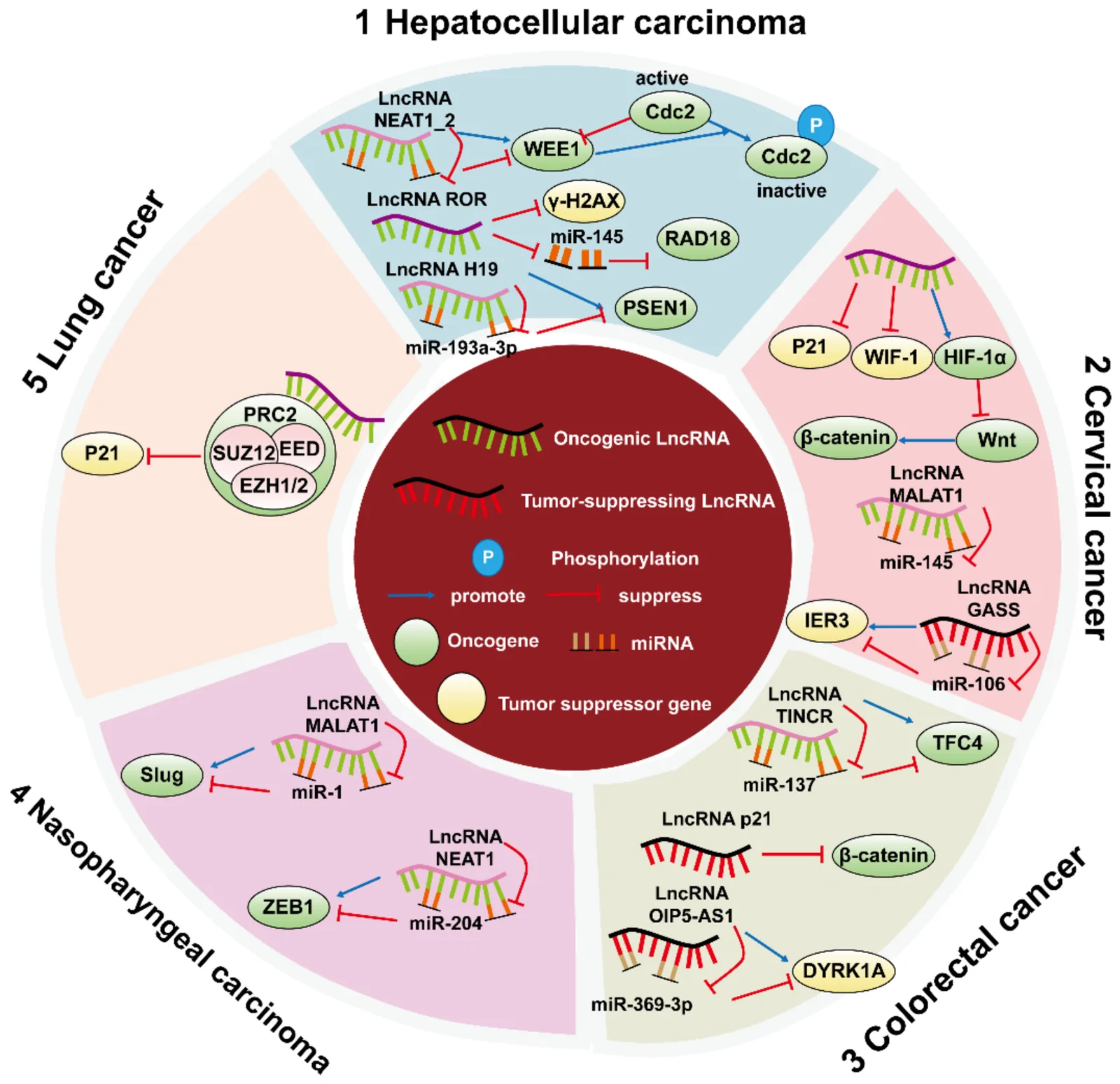
The dual role of non-coding RNA in cancer. The ncRNAs were selected to represent the spectrum of context-dependent oncogenic and tumor-suppressive functions validated across multiple cancer types. Selection criteria included: (1) demonstration of dual functional roles in different cancer contexts; (2) validation by independent research groups; (3) mechanistic insight into context-dependent activity; (4) clinical or therapeutic relevance.

The functional annotation of ncRNAs as “oncogenic” or “tumor-suppressive” is increasingly recognized as an oversimplification. ncRNA function exhibits spatiotemporal plasticity dictated by cell type, tumor stage, microenvironment, and even subcellular localization (Figure 6).

Cell-type dependency: miR-29b is a tumor suppressor in AML, targeting DNMT3A/3B and inhibiting the DNA hypermethylation [70], but it is a metastasis promoter in breast cancer targeting PTEN and activating PI3K/AKT [174]. Likewise, lncRNA MALAT1 is oncogenic in lung and liver cancers while it has been shown to be metastasis suppressive in certain breast cancer subtypes in a context-dependent manner by sponging miRNAs [175]. Tumor stage and tumor microenvironment. Exosomal miR-21 from stromal fibroblasts inhibits the initiation of CRC by targeting COX-2 in early stage. At a late stage, tumor-derived exosomal miR-21 induces M2 immunosuppressive phenotype of macrophages that promotes metastasis [176]. This stage-switching behavior highlights the dynamic nature of ncRNA function, which changes as the tumor progresses. Subcellular localization: The same lncRNA can have opposite effect based on its spatial distribution. The nuclear MALAT1 regulates alternative splicing of oncogenic transcripts [177], while cytoplasmic MALAT1 sponges miR-101 and derepresses EZH2 [178]. circFOXO3 functions tumor-suppressively when it resides in the cytoplasm, but can act as a growth-promoting factor when it remains in the nucleus [178]. Important implications for therapy: The duality of ncRNAs is context-dependent, which makes them challenging targets for therapy. An “oncogenic” ncRNA may have tumor-suppressive activities in certain cell types, and an ASO or siRNA that is used to inhibit the ncRNA may be interfering with this tumor-suppressive activity. However, in some situations, miRNA replacement therapy might prove beneficial, while in others, it might be toxic because of off-target effects on normal tissue homeostasis. This requires tumor subtype-specific and compartment-specific delivery strategies and dynamic biomarker monitoring to guide ncRNA-directed interventions.

Table 3 offers a structured synthesis of key ncRNAs, their molecular mechanisms, functional contexts, and current clinical translation status to facilitate comparative analysis of the above-mentioned ncRNAs. Two important principles that have emerged from this survey are: (i) the context-dependent plasticity of ncRNA function, in which the same ncRNA can have opposing effects depending on the cancer type, tumor stage or subcellular localization; (ii) the translational reality gap, as most ncRNA-based therapeutics are still in the preclinical stages and early-phase clinical trials have faced significant safety and/or efficacy barriers. The clinical development of ncRNA therapeutics has faced some significant achievements and some valuable failures (Table 3). MRX34 (NCT01829971) is the first-in-class miRNA therapeutic to be tested in the clinic, as well as a liposomal miR-34a mimic. The phase I trial was considered to be tolerable with some indications of anti-tumor activity in patients with advanced solid tumors, but development was discontinued because of immune-related adverse events, including two patients with cytokine release syndrome (CRS) [179]. These results highlighted the need to develop optimized dose-scheduling strategies and delivery vehicles for miRNA mimics. A separate study saw let-7 mimics (MRX71, NCT02369198) evaluated in phase I for non-small cell lung cancer, with results to be reported. In the meantime, cobomarsen, an antisense oligonucleotide (ASO) with locked nucleic acid (LNA) modifications targeting miR-155, has a favorable safety profile in phase 1/2 trials for cutaneous T-cell lymphoma (NCT02580552) and diffuse large B-cell (DLBCL) (NCT02220842) and showed evidence of target engagement and preliminary clinical activity [180]. The continued development of cobomarsen underscores the potential of LNA chemistry to improve the pharmacokinetic properties and reduce off-target effects compared with first generation oligonucleotides. Overall, these trials have provided valuable lessons to be learned: (i) for in vivo stability and target engagement, chemical modifications (e.g., LNA, 2’-O-methylation) are required; (ii) delivery systems need to balance tumor targeting with immunogenicity; (iii) patient stratification by ncRNA expression signatures may increase therapeutic response rates. These lessons are used to inform the design of next-generation ncRNA therapeutics outlined below.

**Figure 7:**
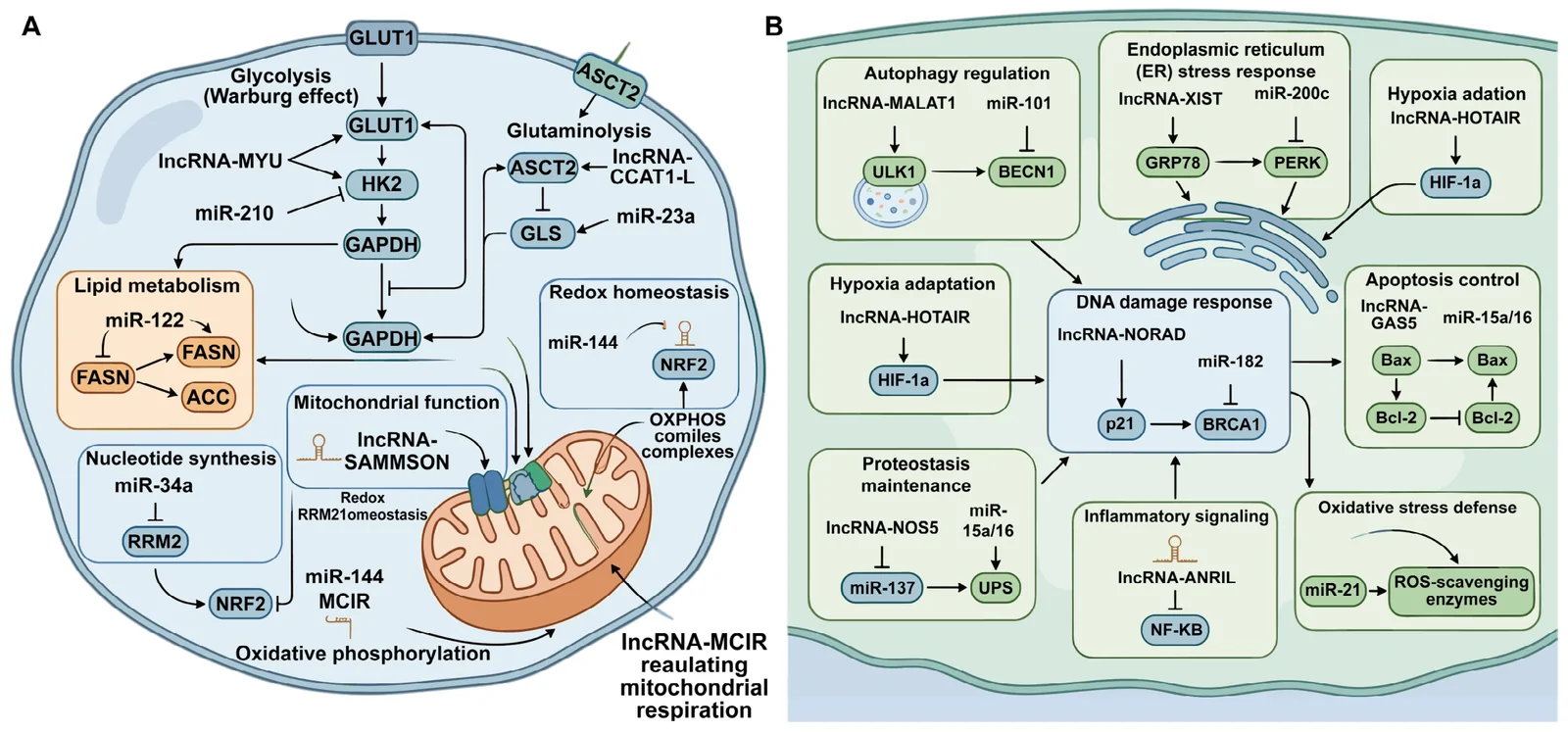
Integrative overview of the functions of ncRNA in cancer metabolism and cellular homeostasis. (**A**) Cancer metabolic reprogramming: ncRNAs control several metabolic pathways in cancer cells, such as glycolysis (Warburg effect), glutaminolysis, lipid metabolism, mitochondrial function, nucleotide synthesis, redox homeostasis and oxidative phosphorylation, which is referred to as metabolic reprogramming. The ncRNA–target pairs are shown; (**B**) Cellular homeostasis and stress response: ncRNAs regulate adaptive response to cellular stress, such as regulation of autophagy, endoplasmic reticulum (ER) stress response, hypoxia adaptation, DNA damage response, apoptosis control, proteostasis maintenance, inflammatory signaling and oxidative stress defense. Regulatory relationship between ncRNAs and the corresponding biological processes are shown as arrow connections.

A few patterns can be discerned from this synthesis. First, miRNAs like miR-34a and let-7 are the most developed therapeutic class, but they have been stymied by immune-related toxicities and tissue-specific delivery issues. Second, lncRNAs are currently mostly at the preclinical stage of ASO development, and hepatotoxicity and renal accumulation are major pharmacokinetic issues. Third, circRNAs and siRNAs are still in their infancy and have only been shown to be effective in localized delivery models with circRNA sponges and allele-specific siRNAs. Of particular interest is the column “context-dependent duality,” which emphasizes that the simplistic classification of oncogenic/tumor-suppressive is not enough for therapeutic design, which is often overlooked in previous reviews (Figure 7). 

## 4. Therapeutic Innovation: From Molecular to Clinical

### 4.1. Delivery Strategies for ncRNA Therapeutics

Safe and efficient delivery systems are crucial for the clinical translation of ncRNA therapeutics. ncRNAs are large, negatively charged hydrophilic molecules that are not able to cross freely cellular membranes, unlike small-molecule drugs. Therefore, various types of delivery strategies have been created to address these challenges. Among the most clinically advanced delivery platform for ncRNA therapeutics are lipid nanoparticles [181]. This approach has been proven successful with the LNP-formulated siRNA (patisiran, Onpattro) and mRNA vaccines (Comirnaty, Spikevax). In the case of ncRNA, LNPs deliver therapeutic oligonucleotides by means of electrostatic interaction, in which the therapeutic oligonucleotides are protected from nuclease degradation and are able to escape from endosomes [182]. The lipid composition (ionizable cationic lipids, helper phospholipids, cholesterol, PEG-lipids), particle size (usually 50–100 nm) and surface charge are key parameters that influence the efficacy of LNP. Recent developments include the creation of organ-selective LNPs via tuning of the ionizable lipid pKa and PEG-lipid ratio, which can be used to target the liver, spleen, or lungs [183]. Liver-targeted delivery of ncRNA therapeutics has become a very successful approach using N-acetylgalactosamine (GalNAc) conjugation. GalNAc has a high affinity for the asialoglycoprotein receptor (ASGPR) which is highly expressed on hepatocytes [184]. This strategy has been successfully implemented in FDA-approved siRNA drugs (givosiran, lumasiran, inclisiran) and is being adopted for miRNA and antagomir delivery. The multivalent presentation of GalNAc (usually triantennary) allows for subcutaneous administration, high hepatocyte uptake and long duration of action [185]. The endogenous extracellular vesicles (exosomes) are vesicles ranging in sizes from 30 to 150 nm, which are very advantageous for the delivery of ncRNA due to their low immunogenicity, intrinsic targeting capabilities and the ability to cross biological barriers, such as the blood–brain barrier [186]. Therapeutic ncRNAs can be loaded into exosomes by parental cell engineering, electroporation or chemical transfection. Engineered exosomes with tumor-targeting ligands (e.g., iRGD, EGFR nanobodies) on their surface have recently been used for the delivery of miRNA mimics [187]. But there are still issues regarding the scalability, standardization and regulatory approval routes of exosomes [187]. DNA or RNA aptamers are short, single-stranded nucleic acid molecules that will bind a target protein with high affinity and can be used as therapeutic agents and targeting ligands. Targeted delivery of a therapeutic ncRNA can be achieved by conjugating it to a cell-specific aptamer, creating an aptamer–ncRNA chimera. For instance, aptamers have been successfully used to deliver antagomirs and siRNAs to prostate cancer and glioblastoma cells, respectively, targeting prostate-specific membrane antigen (PSMA) and transferrin receptor (TfR). The safety profile of ncRNA therapeutics is affected by several factors such as chemical modifications, delivery vehicles, and off-target effects [188]. Chemical modification including 2’-O-methylation, 2’-fluoro, and phosphorothioate backbones to improve nuclease resistance and pharmacokinetic properties may increase immunogenicity [189]. Partial sequence matching can result in unintended suppression of mRNA, complementarity-dependent off-target effects that pose a substantial safety concern. Additionally, delivery vehicles—particularly cationic lipids and polymers—can induce inflammatory responses, complement activation, and hepatotoxicity [190]. The tissue distribution, plasma half-life, and clearance mechanisms of ncRNA therapeutics are sensitive to the delivery strategy and need to be optimized for the specific therapeutic modality. Table 4 summarizes all known clinical trials testing ncRNA-targeted therapeutics, the delivery methods used, the major findings, and the status of the trials [191].

**Figure 8:**
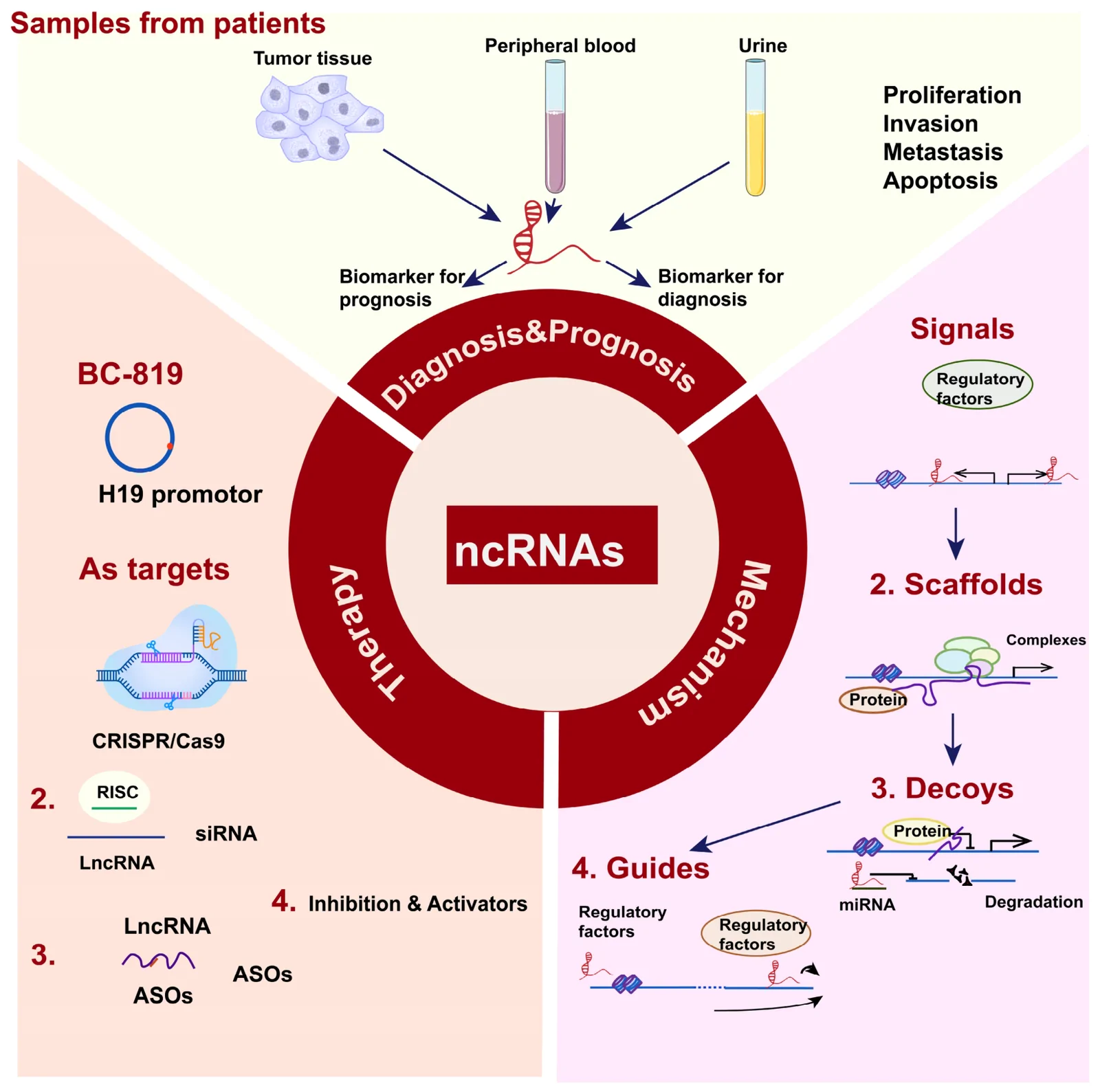
Therapeutic landscape of ncRNAs in cancer. The diagram illustrates the continuum from ncRNA-based diagnosis (liquid biopsy biomarkers, including circulating miRNAs, exosomal ncRNAs, lncRNA panels, and circRNA stability advantages) to mechanistic understanding (oncogenic vs. tumor-suppressive functions, epigenetic regulation, metabolic reprogramming, and context-dependent duality) to therapeutic intervention (miRNA mimics, antagomirs/ASOs/LNA, siRNA/RNAi, circRNA sponges, and CRISPR-Cas13d gene editing). Delivery strategies are indicated: Lipid nanoparticles, GalNAc conjugation, engineered exosomes, aptamer-targeted, direct conjugation. Clinical trial status: MRX34/miR-34a Phase I discontinued, cobomarsen/miR-155 ASO Phase I/II active, miravirsen/miR-122 ASO Phase II completed, TargomiRs/miR-16 Phase I ongoing. Bidirectional arrows emphasize the translational feedback loop between clinical outcomes and mechanistic research.

The ncRNAs listed in Table 4 are representative of a general trend: preclinical efficacy is often strong, but clinical translation is hindered by delivery specificity, off-target immune activation, and pharmacokinetic limitations, as described below. Three main strategies are now in clinical development: (i) loss of function inhibition, (ii) gain of function restoration, and (iii) delivery vector engineering. ASOs and siRNAs: The MALAT1 ASOs were shown to decrease the metastatic burden by 60% in preclinical breast cancer models [192]. However, hepatotoxicity and renal accumulation were observed in phase I trials and led to the development of liver-targeting by GalNAc conjugation [193]. Objective responses were seen in phase II trials of pancreatic cancer with siRNA-mediated targeting, but the local delivery approach restricts its use in cases of metastatic disease [194]. miRNA mimics: MRX34 mimics miR-34a and is formulated as a liposomal miR-34a mimic; it was the first ncRNA therapeutic to enter oncology trials. Phase I was dose-escalated and target modulation was confirmed, but immune-related adverse events (cytokine release syndrome, severe fatigue) caused trial termination [195]. This exemplifies the therapeutic window problem: miRNAs have hundreds of targets, and off-target effects are almost inevitable. Tumor-specific mimics with chemically modified backbones are currently being evaluated. Four interrelated challenges for clinical translation of ncRNA therapeutics exist: (i) Stability and immunogenicity: Unmodified ncRNAs quickly degrade in serum and induce innate immune activation via TLR7/8. Chemical modifications increase stability, but could decrease affinity for the target and increase hepatotoxicity [196]. (ii) Tissue specificity and biodistribution: Despite the use of GalNAc, the extrahepatic delivery to solid tumors is still inefficient. Tumor penetration is further limited by high interstitial pressure and dense extracellular matrix. The tumor uptake of nanoparticle based systems is ~1–2% and most of the nanoparticles are deposited in the liver and spleen [197]. (iii) Off-target effects and sequence homology: Both miRNAs and siRNAs can produce unintended silencing of targets with partial seed-sequence complementarity and cause toxic phenotypes. For instance, miR-122 mimic (miravirsen) for hepatitis C (HCV) resulted in upregulation of lipogenic genes and hepatic steatosis in preclinical models [198]. (iv) Production and price. Chemically modified oligonucleotides are not widely available and cost $10,000–$50,000 per gram for large-scale GMP synthesis. Recent innovations are designed to address these challenges. Exosomes are vehicles that can be harnessed for delivery using their own biocompatibility and tumor-targeting ability. iRGD peptides were engineered onto exosomes that delivered siRNA against RAD51 with 5-fold greater tumor accumulation than liposomal counterparts [199,200]. In vivo delivery of CRISPR/Cas13d systems is challenging, but these systems can edit the transcriptome with higher specificity than RNAi [201]. The use of artificial intelligence (AI) for guided design to predict off-target effects and optimize chemical modifications: Although ncRNA therapeutics are a paradigm shift from protein-targeted drugs, the field needs to move from proof-of-concept to precision dosing based on mechanism. Early phase trials have shown that biological activity in cell lines is not necessarily a predictor of clinical activity if pharmacokinetic and immunological challenges are not addressed. Recent advances have further improved the understanding of ncRNA topology and therapeutic delivery [202]. For example, clarified the structural mechanism of lncRNA–protein phase separation in the regulation of transcription, and showed the design of tumor-penetrating peptide (TPP)-conjugated exosomes for siRNA delivery with lowered immunogenicity [203]. To achieve success in the future, multi-omics patient stratification, advanced delivery systems, and comprehensive toxicological profiling (Figure 8) will be essential. A comprehensive summary of clinical trials evaluating ncRNA-targeted therapeutics is provided in Table 4. 

**Table 4:** Summary of clinical trials evaluating ncRNA-targeted therapeutics in cancer. CTCL, cutaneous T-cell lymphoma; LNA, locked nucleic acid; ASO, antisense oligonucleotide.

Trial_ID	ncRNA_Target	Therapeutic_Modality	Cancer_Type	Phase	Delivery_Strategy	Key_Findings	Status	Reference
NCT01829971	miR-34a	miRNA mimic (liposomal)	Solid tumors (multiple)	I	SMARTICLES liposomes	Dose-limiting cytokine release syndrome; 1 partial response observed	Discontinued	[189]
NCT02580552	miR-155	LNA-antisense (cobomarsen)	CTCL/mycosis fungoides	I/II	Systemic (LNA-modified, naked)	Target engagement confirmed; favorable safety profile; no DLTs	Active (recruiting)	[204]
NCT01227417	miR-10b	Antagomir (locked nucleic acid)	Solid tumors (multiple)	I	Intravenous (naked)	Good tolerability; evidence of miR-10b target modulation in serum	Completed	[205]
NCT02426892	miR-21	LNA-antisense	Malignant pleural mesothelioma	I	Intravenous	Completed; demonstrated tolerability in mesothelioma patients	Completed	[206]
NCT02120456	miR-122	LNA-antisense (miravirsen)	Hepatocellular carcinoma	II	Subcutaneous (GalNAc-conjugated)	Dose-dependent reduction in miR-122; well tolerated up to highest dose	Completed	[207]
NCT02862145	miR-16 (in EBV)	miRNA mimic (EDV nanocell)	Nasopharyngeal carcinoma	I	Intrapleural (EDV nanocells)	Completed; intrapleural delivery feasible; disease stabilization in 5/12	Completed	[205]
NCT03460258	miR-200c	miRNA mimic (liposomal)	Metastatic cancer	I	Liposomal formulation	Ongoing; formulation optimization for metastatic settings	Active (not recruiting)	[208]
NCT00005978	BCL-2 (ASO)	ASO (oblimersen sodium)	Melanoma (stage IV)	III	Intravenous infusion	Improved progression-free survival vs. dacarbazine (6.4 vs. 5.7 mo)	Completed	[209]

### 4.2. Artificial Intelligence in ncRNA Therapeutic Design

The use of artificial intelligence (AI) in the design of ncRNA therapeutics is rapidly expanding, with the potential to revolutionize sequence optimization, target prediction, and delivery system engineering [210]. Machine learning algorithms, such as deep neural networks and transformer architectures, have been used to predict optimal ASO/siRNA sequences, model off-target effects, and design chemical modifications [210]. However, recent advances in the field, including the structure prediction capabilities of Google DeepMind’s AlphaFold extensions and specialized RNA folding models like the RNA structure prediction model, have paved the way for structure-guided design of ncRNA therapeutics [211]. AI-based methods have demonstrated great potential in the following areas: (i) prediction of miRNA–target interaction with higher accuracy than seed-based methods, taking into account accessibility, site conservation and structural context; (ii) optimization of LNA/2’-F modification pattern to improve binding affinity whilst reducing immunogenicity; (iii) design of targeted nanomedicine delivery systems by high-throughput screening and generative models; (iv) integrative analysis of multi-omics data and clinical outcome to identify patient subpopulations most likely to benefit from ncRNA-based therapies [210]. However, there are still hurdles to be addressed, such as experimental validation of AI predictions, interpretability of deep learning models, and integrating AI-designed candidates into current drug development pipelines. The future should involve the creation of extensive training datasets, standardization and benchmarking, and interdisciplinary collaboration between computational scientists and experimental biologists to maximize the potential of AI in the development of ncRNA therapeutics.

## 5. Conclusions

This review suggests that ncRNAs are not mere individual regulators but are hierarchical coordinators of cancer systems, spanning across the epigenetic, transcriptional, post-transcriptional, signaling and metabolic levels. The mechanistic principles and the translational implications of ncRNA biology have been critically assessed. Unresolved Questions: Three priorities arise for the coming decade: (i) Contextual mapping: How do ncRNA functions change with tumor stages and microenvironments? To address this plasticity, single-cell multi-omics and spatial transcriptomics will be key. (ii) Mechanistic integration: How does ncRNA-directed chromatin remodeling, metabolic rewiring, and immune evasion work together to maintain tumor heterogeneity? To uncover these cross-layer interactions, systems biology approaches are required. (iii) Clinical feasibility: Can the delivery technologies be used to modulate ncRNAs in a tumor-specific manner without causing systemic toxicity? The combination of engineered exosomes, CRISPR-Cas13d and AI-guided target prediction appears to be a promising direction going forward, but there is still a lack of robust phase III data. Final Reflection: The complexity of the non-coding genome is reflected in the fact that ‘junk DNA’ can be turned into actionable therapeutic targets. But the translation from molecular discovery to clinical benefit is still a long way. Future reviews and research need to shift from descriptive cataloging to quantitative, context-based and clinically oriented frameworks. It is only then that the promise of ncRNA biology in precision oncology can be realized.

## Figures and Tables

**Table 1:** Established versus emerging ncRNA mechanisms in cancer.

Mechanism	Category	Key_Evidence	Validation_Status	Clinical_Relevance
lncRNA-guided PRC2 recruitment to chromatin	Established	HOTAIR/ANRIL ChIP-seq; functional rescue; clinical correlation (>50 studies)	High	ASO clinical trials (HOTAIR)
miRNA-mediated DNMT regulation (miR-29 family)	Established	miR-29/DNMT3B luciferase assays; patient sample validation (>30 studies)	High	miR-29 mimics in development
lncRNA-mediated histone modification targeting	Established	Multiple lncRNA-protein interaction studies; histone mark profiling (>20 studies)	High	Epigenetic drug combinations
miRNA seed-based mRNA targeting (3′ UTR)	Established	Conserved seed sequences; transcriptome-wide validation; AGO HITS-CLIP (>1000 studies)	High	miRNA replacement therapy (MRX34)
circRNA miRNA sponging (ciRS-7/CDR1as)	Established	ciRS-7 >70 miR-7 binding sites; luciferase; in vivo validation (>20 studies)	High	circRNA biomarkers in trials
circRNA modulation of chromatin accessibility	Emerging	Preliminary circRNA-chromatin interaction data; limited functional studies (2–3)	Low	Preclinical only
ncRNA regulation of 3D genome organization	Emerging	Hi-C correlation analyses; proposed models; few mechanistic studies (1–2)	Low	Hypothesis stage
miRNA regulation of chromatin remodelers	Emerging	Bioinformatic predictions; limited in vivo validation (5–10 studies)	Moderate	Preclinical validation
lncRNA scaffolding of signaling complexes	Established	HOTAIR-PRC2-LSD1 complex; MALAT1-SR protein interactions (>20 studies)	High	ASO/siRNA development
circRNA translation into functional peptides	Emerging	circ-ZNF609 translation demonstrated; limited cancer relevance (3–5 studies)	Moderate	Emerging therapeutic target

**Table 2:** circRNA-mediated regulation of signaling pathways in cancer.

Pathway	circRNA	Target/Mechanism	Cancer Type	Functional Outcome	Validation
Wnt/β-catenin	circ-β-catenin	Sponges miR-200a → activates β-catenin	Colorectal cancer	Promotes proliferation and invasion	Luciferase + rescue
Wnt/β-catenin	circCCDC66	Sponges miR-33b/miR-93 → activates Wnt/β-catenin	Colorectal cancer	Promotes tumor growth and metastasis	Luciferase + rescue + in vivo
Wnt/β-catenin	circ_002454	Sponges miR-125b → upregulates WNT5A	Gastric cancer	Enhances stemness and chemoresistance	Luciferase + patient samples
PI3K/AKT	circHIPK3	Sponges miR-124 → activates PI3K/AKT/mTOR	Glioma, breast cancer	Promotes cell proliferation and migration	Luciferase + rescue + mouse
PI3K/AKT	circAGFG1	Sponges miR-433-3p → upregulates FGF2	Nasopharyngeal carcinoma	Promotes metastasis and EMT	Luciferase + rescue
PI3K/AKT	circPIP5K1A	Sponges miR-600 → activates PI3K/AKT	Lung cancer	Promotes proliferation and glycolysis	Luciferase + rescue + in vivo
PI3K/AKT	circ-SHKBP1	Sponges miR-582-3p → activates PI3K/AKT	Glioma	Promotes angiogenesis and tumor growth	Luciferase + rescue
NF-κB	circRNA_000203	Sponges miR-26b-5p → activates NF-κB/CTGF	Cardiac fibrosis	Promotes fibrosis (NF-κB activation)	Luciferase + rescue
NF-κB	circANRIL	Binds PES1 → activates p53/NF-κB	Atherosclerosis	Regulates inflammatory response	Pull-down + rescue
NF-κB	circ_0001806	Sponges miR-218 → activates NF-κB pathway	Osteosarcoma	Promotes proliferation and migration	Luciferase + rescue

**Table 3:** Summary of ncRNA–target interactions with demonstrated context-dependent functional duality, wherein the same ncRNA can exert either oncogenic or tumor-suppressive effects depending on the cancer type, cellular microenvironment, or disease stage. The “Functional Role” column indicates the dual nature of each interaction, highlighting the necessity of context-specific analysis when evaluating ncRNA-based therapeutic strategies.

ncRNA	Type	Cancer Type(s)	Molecular Mechanism	Functional Role	Clinical Stage
miR-34a	miRNA	Hepatocellular carcinoma, NSCLC, mesothelioma, multiple solid tumors	Direct targeting of c-MYC, BCL-2, CDK6, PD-L1; p53-driven transcriptional activation; induces apoptosis and cell-cycle arrest	Tumor-suppressive context-dependent toxicity in normal tissues)	Phase I (discontinued due to immune-related adverse events: MRX34, NCT01829971); Phase I/II ongoing with tumor-specific promoters (NCT05605053)
miR-15a/16-1 cluster	miRNA	Chronic lymphocytic leukemia (CLL), mantle cell lymphoma	Deletion at 13q14.3 leads to loss of miR-15a/16-1; derepression of BCL-2 anti-apoptotic axis; promotes cell survival	Tumor-suppressive (deletion = oncogenic)	Preclinical; candidate for replacement therapy in 13q-deleted CLL
let-7 family	miRNA	Lung adenocarcinoma, breast cancer, ovarian cancer	Targets RAS (MAPK pathway), HMGA2 (chromatin remodeling), CDC25A/CDK6; blocks proliferation and stemness maintenance	Tumor-suppressive; low expression correlates with cancer stem cell properties	Preclinical; miRNA mimic formulations under development (LNA-modified let-7b)
miR-21	miRNA	Colorectal cancer, breast cancer, glioblastoma, pancreatic cancer	Targets PTEN (PI3K/AKT negative regulator), PDCD4, TPM1; promotes proliferation, invasion, 5-FU chemoresistance; exosomal transfer to stromal cells induces M2 macrophage polarization	Oncogenic (stage-dependent: stromal-derived miR-21 may suppress early tumorigenesis)	Preclinical; antagomir (RG-125/AZD4076) tested in Phase I for fibrotic disease, repurposing for cancer under evaluation
miR-221/222	miRNA	Breast cancer (ER^+^), hepatocellular carcinoma, glioblastoma	Targets p27^Kip1, p57^Kip2, ERα; drives G1/S transition; induces tamoxifen resistance via EMT activation	Oncogenic; enriched in cancer stem cells	Preclinical; locked nucleic acid (LNA) antagomirs in development
miR-155	miRNA	B-cell lymphoma, breast cancer, lung cancer	Targets SOCS1 and C/EBPβ; activates JAK/STAT and NF-κB signaling; promotes inflammation-driven tumorigenesis	Oncogenic (immune context-dependent: required for normal B-cell function)	Phase I (cobomarsen/MRG-106, NCT02580552) for cutaneous T-cell lymphoma; discontinued for solid tumors due to on-target immune suppression
miR-29b	miRNA	Acute myeloid leukemia (AML), breast cancer, cholangiocarcinoma	AML: targets DNMT3A/3B, reduces DNA hypermethylation, restores tumor suppressor expression. Breast cancer: targets PTEN, activates PI3K/AKT and promotes metastasis	Context-dependent duality: tumor-suppressive in hematologic malignancies; oncogenic in solid tumors via microenvironment crosstalk	Preclinical; lipid nanoparticle encapsulation tested in AML xenografts
HOTAIR	lncRNA	Breast cancer, hepatocellular carcinoma, colorectal cancer, gastrointestinal stromal tumors	5′ domain binds PRC2 (EZH2, catalyzing H3K27me3); 3′ domain recruits LSD1/CoREST/HDAC (H3K4me2 demethylation); bivalent silencing of tumor suppressor loci; promotes chemoresistance and metastasis	Oncogenic; high expression correlates with poor prognosis and metastasis	Preclinical; ASO (IONIS-MALAT1Rx analog) tested in breast cancer models; hepatotoxicity observed at high doses in vivo
MALAT1	lncRNA	Lung adenocarcinoma, hepatocellular carcinoma, breast cancer (subtype-specific), bladder cancer	Nuclear: regulates alternative splicing via SR proteins (phosphorylation control). Cytoplasmic: sponges miR-101-3p/EZH2 and miR-32-5p/HK2; binds VDAC1 to anchor HK2 to mitochondria (Warburg effect); enhances mTOR-mediated TCF7L2 translation	Context-dependent duality: oncogenic in lung/liver; tumor-suppressive in specific breast cancer subtypes via miRNA sponging	Preclinical; ASO gapmers reduce metastatic burden by 60% in mouse models; renal accumulation limits systemic delivery
MEG3	lncRNA	Hepatocellular carcinoma, glioma, pheochromocytoma, multiple myeloma	Activates p53-dependent apoptotic signaling by binding MDM2 and preventing p53 ubiquitination/degradation; also recruits PRC2 to silence growth-promoting genes; imprinted lncRNA lost via promoter hypermethylation in cancers	Tumor-suppressive; epigenetic silencing is a common oncogenic mechanism	Preclinical; DNA-demethylating agents (decitabine) restore MEG3 expression in vitro

## Data Availability

No new data were created or analyzed in this study. Data sharing is not applicable to this article.
